# Novel antigen-presenting cell imparts T_reg_-dependent tolerance to gut microbiota

**DOI:** 10.1038/s41586-022-05309-5

**Published:** 2022-09-07

**Authors:** Blossom Akagbosu, Zakieh Tayyebi, Gayathri Shibu, Yoselin A. Paucar Iza, Deeksha Deep, Yollanda Franco Parisotto, Logan Fisher, H. Amalia Pasolli, Valentin Thevin, Rasa Elmentaite, Maximilian Knott, Saskia Hemmers, Lorenz Jahn, Christin Friedrich, Jacob Verter, Zhong-Min Wang, Marcel van den Brink, Georg Gasteiger, Thomas G. P. Grünewald, Julien C. Marie, Christina Leslie, Alexander Y. Rudensky, Chrysothemis C. Brown

**Affiliations:** 1 Immuno-Oncology, Human Oncology and Pathogenesis Program, Memorial Sloan Kettering Cancer Center, New York, USA; 2 Computational and Systems Biology Program, Memorial Sloan Kettering Cancer Center, New York, NY USA; 3 Tri-Institutional Program in Computational Biology and Medicine, Weill Cornell Graduate School, New York, NY USA; 4 Immunology and Microbial Pathogenesis Program, Weill Cornell Medicine Graduate School of Medical Sciences, New York, NY USA; 5 Howard Hughes Medical Institute and Immunology Program, Sloan Kettering Institute and Ludwig Center at Memorial Sloan Kettering Cancer Center, New York, NY USA; 6 Tri-Institutional MD-PhD Program, Weill Cornell Medicine, The Rockefeller University and Memorial Sloan Kettering Cancer Center, New York, NY USA; 7 Electron Microscopy Resource Center, The Rockefeller University, New York, NY USA; 8 Tumor Escape Resistance Immunity Department, CRCL, INSERM U1052, CNRS 5286, Centre Léon Bérard, Université de Lyon, Lyon, France; 9 Equipe Labellisée Ligue Nationale contre le Cancer, Lyon, France; 10 Wellcome Sanger Institute, Wellcome Genome Campus, Hinxton UK; 11 Institute of PathologyFaculty of Medicine, LMU Munich, Munich, Germany; 12 Department of Immunology, Memorial Sloan Kettering Cancer Center, New York, NY USA; 13 Würzburg Institute of Systems Immunology, Max Planck Research Group, Julius-Maximilians-Universität Würzburg, Würzburg, Germany; 14 Department of Medicine, Memorial Sloan Kettering Cancer Center, New York, NY USA; 15 Hopp—Children’s Cancer Center Heidelberg (KiTZ), Heidelberg, Germany; 16 Division of Translational Pediatric Sarcoma Research, German Cancer Research Center (DKFZ), German Cancer Consortium (DKTK), Heidelberg, Germany; 17 Institute of Pathology, Heidelberg University Hospital, Heidelberg, Germany; 18 Department of Pediatrics, Memorial Sloan Kettering Cancer Center, New York, NY USA; 19 Parker Institute for Cancer Immunotherapy, Memorial Sloan Kettering Cancer Center, New York, NY USA; 20 Present Address: Department of Immunology, Duke University, Durham, NC USA

**Keywords:** Mucosal immunology, Immune tolerance

## Abstract

Establishing and maintaining tolerance to self-antigens or innocuous foreign antigens is vital for the preservation of organismal health. Within the thymus, medullary thymic epithelial cells (mTECs) expressing autoimmune regulator (AIRE) have a critical role in self-tolerance through deletion of autoreactive T cells and promotion of thymic regulatory T (T_reg_) cell development^1–4^. Within weeks of birth, a separate wave of T_reg_ cell differentiation occurs in the periphery upon exposure to antigens derived from the diet and commensal microbiota^5–8^, yet the cell types responsible for the generation of peripheral T_reg_ (pT_reg_) cells have not been identified. Here we describe the identification of a class of RORγt^+^ antigen-presenting cells called Thetis cells, with transcriptional features of both mTECs and dendritic cells, comprising four major sub-groups (TC I–TC IV). We uncover a developmental wave of Thetis cells within intestinal lymph nodes during a critical window in early life, coinciding with the wave of pT_reg_ cell differentiation. Whereas TC I and TC III expressed the signature mTEC nuclear factor AIRE, TC IV lacked AIRE expression and was enriched for molecules required for pT_reg_ generation, including the TGF-β-activating integrin αvβ8. Loss of either major histocompatibility complex class II (MHCII) or ITGB8 by Thetis cells led to a profound impairment in intestinal pT_reg_ differentiation, with ensuing colitis. By contrast, MHCII expression by RORγt^+^ group 3 innate lymphoid cells (ILC3) and classical dendritic cells was neither sufficient nor required for pT_reg_ generation, further implicating TC IV as the tolerogenic RORγt^+^ antigen-presenting cell with an essential function in early life. Our studies reveal parallel pathways for the establishment of tolerance to self and foreign antigens in the thymus and periphery, respectively, marked by the involvement of shared cellular and transcriptional programmes.

## Abstract (web summary)

Single-cell transcriptomic and epigenetic analysis has enabled the identification of Thetis cells, a class of RORγt^+^ antigen-presenting cells with a key role in the differentiation of commensal microbiota-induced peripheral regulatory T cells.

## Main

In the thymus, a distinct lineage of epithelial cells establishes tolerance to self-antigens through deletion of autoreactive T cells and promotion of T_reg_ cell differentiation^1–4^. These functions of mTECs are mediated in part through the expression of AIRE, which regulates the ectopic expression of tissue-restricted antigens^1^. Another major site of tolerance induction resides within intestinal lymphoid tissue, where an infant’s developing immune system is exposed to many new dietary components and colonizing microbes upon weaning. Establishing a harmonious host–microbiota relationship in this early life developmental window is critical to prevent later onset of immune-mediated disorders^7,9^. Central to the establishment of tolerance to intestinal microbes is the differentiation of naive T cells to peripherally generated T_reg_ (pT_reg_) cells upon encounter with commensal-derived antigens^5,6,10,11^. Yet the identity of the antigen-presenting cell (APC) that promotes pT_reg_ cell differentiation is not known. The narrow time window for establishing intestinal immune homeostasis suggests the presence of a developmentally restricted tolerogenic APC within the neonatal intestinal niche.

## RORγt^+ ^APCs promote pT_reg_ differentiation 

**Fig. 1:**
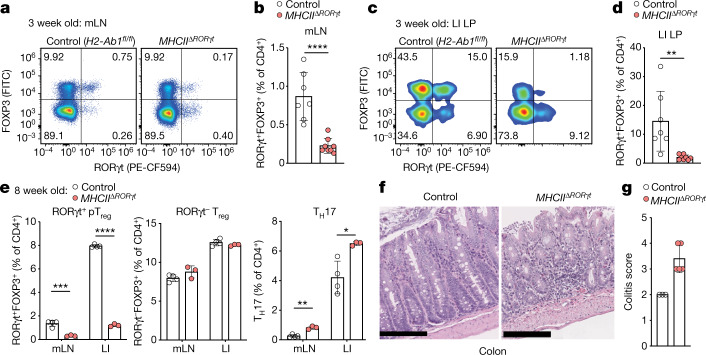
RORγt^+^ APCs promote pT_reg_ differentiation and intestinal tolerance during early life. **a**–**d**, Flow cytometry of RORγt and FOXP3-expressing CD4^+^ T cell subsets (**a**,**c**) and summary graphs (**b**,**d**) for frequencies of pT_reg_ (RORγt^+^FOXP3^+^) cells in mLN (**a**,**b**) and large intestine lamina propria (LI LP) (**c**,**d**) of 3-week-old *MHCII*^*ΔRORγt*^ (*n* = 7) and control (*H2-Ab1*^*fl/fl*^) (*n* = 8) mice. **e**, Eight-week-old *MHCII*^*ΔRORγt*^ (*n* = 4) and control (*n* = 3) mice were analysed for frequencies of pT_reg_ (RORγt^+^FOXP3^+^) cells, RORγt^–^ T_reg_ cells and T_H_17 (FOXP3^−^RORγt^+^) cells among CD4^+^ T cells in indicated tissues. **f**, Representative haematoxylin and eosin (H&E)-stained sections of colon from *MHCII*^*ΔRORγt*^ and control mice at 12 weeks of age. Scale bars, 200 μm. **g**, Histological colitis score in 12-week-old *MHCII*^*ΔRORγt*^ (*n* = 5) and control (*n* = 3) mice. Data are mean ± s.e.m. Each symbol represents an individual mouse. Data in **a** are pooled from two independent experiments. Data in **e** are representative of three independent experiments. Two-tailed unpaired *t*-test. *P < 0.05, **P < 0.01, ***P < 0.001 and ***P < 0.0001. Source data

**Extended Data Fig. 1:**
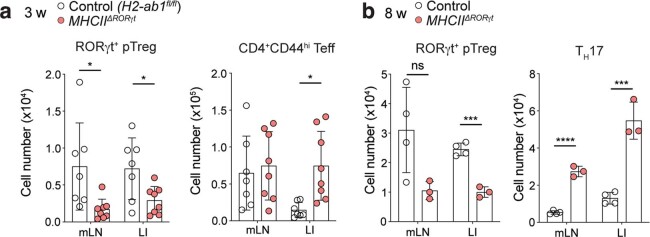
Analysis of pTreg cell generation in mice harboring MHC class II-deficient RORγt^+^ APCs. **a**, Quantification of total pTreg (RORγt^+^Foxp3^+^) and CD4^+^ T_eff_ (Foxp3^–^CD44^hi^) cells in the mesenteric lymph nodes (mLN) and large intestine lamina propria (LI) of 3-week-old *MHCII*^*ΔRORγt*^ and control (*H2-Ab1*^*fl/fl*^) mice (*n* = 7 or 8 mice per group). **b**, pTreg (RORγt^+^Foxp3^+^) and T_H_17 (Foxp3^–^CD44^hi^RORγt^+^) cells in mLN and LI of 8-week-old *MHCII*^*ΔRORγt*^ (*n* = 4) and control (*n* = 3) mice. Data in **a** pooled from two independent experiments. Data in **b** representative of three independent experiments. Error bars: means ± s.e.m. Statistics were calculated by unpaired two-sided *t*-test; *P < 0.05; ***P < 0.01, ****P < 0.0001. Source data

Extra-thymic pT_reg_ cells, distinguished from their thymic counterparts by expression of the orphan nuclear receptor RORγt, arise in mesenteric lymph nodes (mLN) in response to commensal bacterial antigens and have a critical role in suppressing inflammatory immune responses against gut microbes^6,11^. Conversely, mice deficient in MHCII-restricted antigen presentation by RORγt^+^ cells (*MHCII*^*ΔRORγt*^), develop severe intestinal inflammation because they do not establish tolerance to commensal bacteria^12^, suggesting a potential connection between RORγt^+^ APCs and RORγt^+^ pT_reg_ cell generation. To address this possibility, we analysed *MHCII*^*ΔRORγt*^ mice at three weeks of age, when pT_reg_ cells accumulate in the intestine^5,6^. We observed a marked reduction in RORγt^+^ pT_reg_ cells within the mLN and colonic lamina propria, along with an expansion of CD44^hi^ T effector (T_eff_) cells (Fig. 1a–d and Extended Data Fig. 1a). At eight weeks of age, these mice exhibited a sustained, severe reduction in RORγt^+^ pT_reg_ cells along with expansion of colonic T helper 17 (T_H_17) cells (Fig. 1e and Extended Data Fig. 1b), in line with previous studies demonstrating a prominent role for pathobiont-specific RORγt^+^ pT_reg_ cells in suppressing inflammatory T_H_17 cells^11^. Histological analysis demonstrated severe colitis with marked inflammatory cell infiltrate, mucosal ulceration and loss of crypts (Fig. 1f,g), confirming a critical role for RORγt^+^ APCs in preventing dysregulated intestinal immune responses.

**Extended Data Fig. 2:**
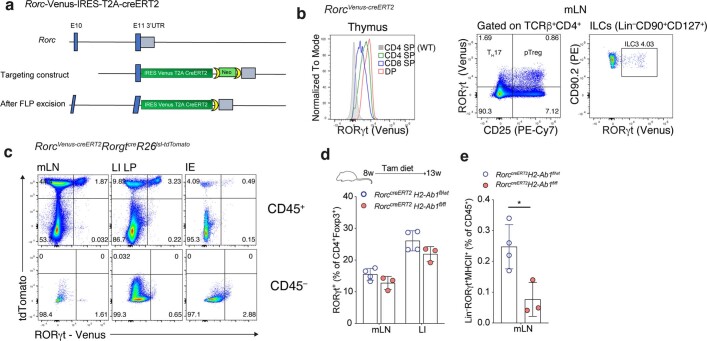
Temporal ablation of MHC Class II on RORγt^+^ APCs. **a**, Targeting strategy for the *Rorc* locus. **b**, Flow cytometry of Venus expression in thymocytes (left) or mLN TCRβ^+^CD4^+^ T cells (middle) and Lin^–^CD90^+^CD127^+^ innate lymphoid cells (ILC; right) isolated from adult mice. **c**, Flow cytometry of mLN, LI and intestinal epithelial (IE) CD45^+^ and CD45^–^ cells in P16 *Rorc* reporter RORγt fate-mapper (*Rorc*^*Venus-creERT2*^*Rorgt*^*cre*^*Rosa26*^*lsl-tdTomato*^) mice. Representative of *n* = 3 mice. **d**-**e**, Frequency of pTreg cells amongst CD4^+^Foxp3^+^ cells in mLN and LI (**d**) or frequency of RORγt^+^ APCs (Lin–RORγt(Venus)^+^MHCII^+^) (**e**) in mLN of *Rorc*^*Venus-creERT2*^*H2-Ab1*^*fl/fl*^ (n = 4) or *Rorc*^*Venus-creERT2*^*H2-Ab1*^*fl/wt*^ (*n* = 3) mice maintained on tamoxifen diet from 8–13 weeks of age. Each symbol represents an individual mouse. Data in **b**–**d** representative of two independent experiments. Error bars: means ± s.e.m.; *P < 0.05; unpaired two-sided *t*-test. Source data

Given previous reports of a developmental window for intestinal immune tolerance^7,8^, we addressed whether the pT_reg_ cell deficit in adult *MHCII*^*ΔRORγt*^ mice reflected a failure to generate pT_reg_ cells in early life or a continuous requirement for RORγt^+^ APC-instructed pT_reg_ cell differentiation. We therefore generated a *Rorc*^*Venus-creERT2*^ allele for identification and temporal manipulation of RORγt^+^ cells (Extended Data Fig. 2a,b). Analysis of *Rorgt*^*cre*^*Rosa26*^*lsl-tdTomato*^*Rorc*^*Venus-creERT2*^ mice confirmed that expression of Venus protein, translated downstream of exon 11, faithfully reflected expression of the RORγt isoform within the mLN and large intestine (Extended Data Fig. 2c). Surprisingly, continuous ablation of MHCII on RORγt^+^ APCs in adult *Rorc*^*Venus-creERT2*^*H2-Ab1*^*fl/fl*^ mice treated with tamoxifen from 8–13 weeks of age resulted in only a modest decrease in pT_reg_ cells within the mLN and LI (Extended Data Fig. 2d,e), indicating a minimal contribution of de novo pT_reg_ cell differentiation to the pT_reg_ cell pool of adult mice with stable microbial communities. Together, these results demonstrate an essential role for an early life RORγt^+^ APC in pT_reg_ cell generation and raise the question of the nature of the tolerogenic RORγt^+^ APC.

## Identification of a novel lineage of RORγt^+^ APCs

**Fig. 2:**
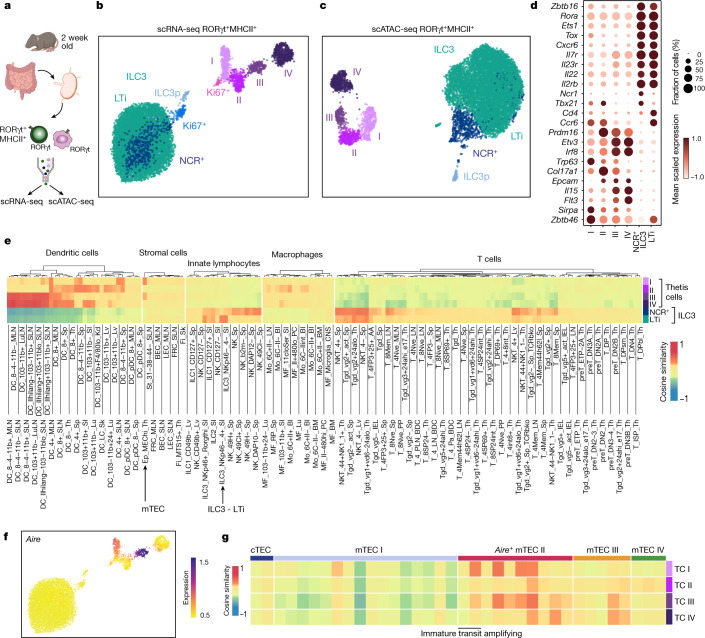
Identification of a novel RORγt^+^ APC lineage. **a**, Schematic of paired scRNA-seq and scATAC-seq of Lin^–^RORγt^+^MHCII^+^ cells from the mLN of 2-week-old *Rorc*^*Venus-creERT2*^ mice (pooled from 16 biological replicates). **b**,**c**, Uniform manifold approximation and projection (UMAP) visualization of 10,145 cells profiled by scRNA-seq (**b**) or scATAC-seq (**c**), coloured by cluster annotation. **d**, Dot plot showing the expression of canonical ILC3 or cluster I–IV marker genes **e**, Similarity between cell types identified in **b** and ImmGen bulk microarray profiles for immune and stromal cells. Cell lineages in which any individual cell type exhibited a cosine similarity greater than 0.25 were included in the visualization. **f**, scRNA-seq UMAP overlaid with imputed expression of *Aire*. **g**, Similarity between Thetis cell subsets in **b** and thymic epithelial subsets from publicly available single-cell transcriptomic data (cTEC; cortical thymic epithelial cell).

**Extended Data Fig. 3:**
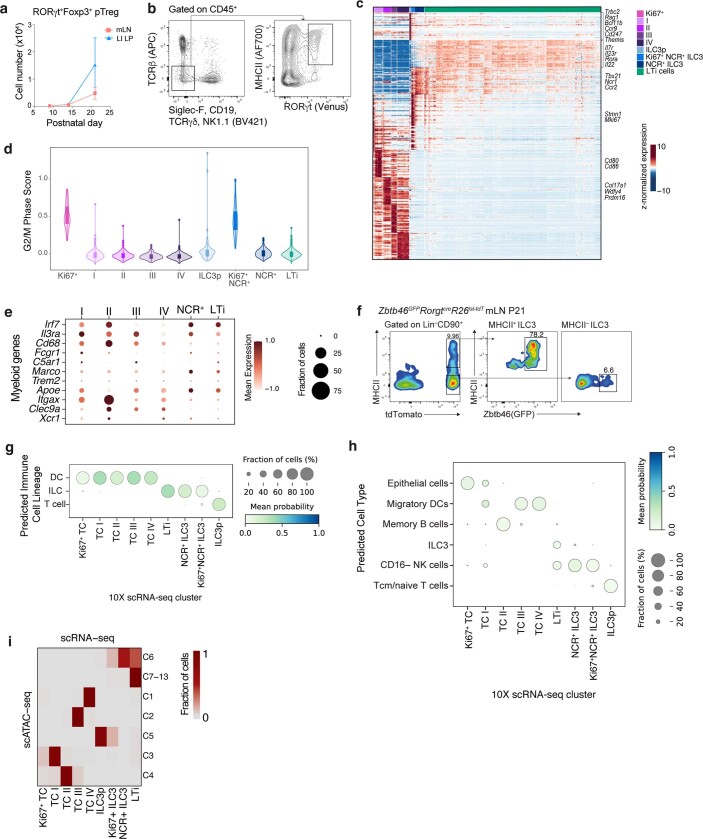
Identification of a novel RORγt^+^ APC lineage. **a**, RORγt^+^Foxp3^+^ pTreg cell numbers in mLN and LI of *Rorc*^*Venus-creERT2*^ mice at indicated postnatal ages (*n* = 3–4 mice per time-point). **b**, Cell sorting scheme for Lin(Siglec-F, TCRβ, TCRγδ, CD19, NK1.1)^–^RORγt(Venus)^+^MHCII^+^ cells. **c**, Heatmap reporting scaled, imputed expression of top differentially expressed genes for each scRNA-seq cluster (one vs the rest, FC > 1.5, *P* < 0.01). **d**, Expression score of cell-cycle genes for each scRNA-seq cluster. **e**, Dot plot showing expression of myeloid genes. **f**, Flow cytometry of Zbtb46 (GFP) expression in ILC3 subsets from mLN of 3-week-old *Zbtb46*^GFP^*Rorgt*^*cre*^*R26*^*lsl-tdTomato*^ mice. Representative of *n* = 4 mice from two independent experiments. **g**-**h**, CellTypist derived cell labels for cell clusters from Fig. 2g, using a broad classification (**g**) or finer cell type annotation (**h**). **i**, Correspondence between cell labels for scATAC-seq and scRNA-seq. Source data

A number of candidate APC types have been suggested to regulate tolerance to the intestinal microbiota, including dendritic cells and MHCII^+^ ILC3s (also known as lymphoid tissue inducer (LTi)-like cells). Among these, the loss of tolerance to commensals in *MHCII*^*ΔRORγt*^ mice has previously been attributed to ILC3s on the assumption that they represent the only RORγt^+^MHCII^+^ cell type^12,13^. However, recent studies have identified RORγt-expressing dendritic cells^14,15^ as well as RORγt^+^AIRE^+^ cells^16^, which were initially described as ‘ILC3-like’ cells but were subsequently shown to more closely resemble dendritic cells^17^. The role of these cells in immune tolerance remains unknown. Critically, the spectrum of RORγt^+^ APCs has not been examined within the mLN at the time when pT_reg_ cells first arise. RORγt^+^ pT_reg_ cells first appeared within the mLN between postnatal day (P)10 and P14, with rapid accumulation thereafter (Extended Data Fig. 3a). We therefore performed paired single-cell RNA sequencing (RNA-seq) and single-cell assay for transposase-accessible chromatin using sequencing (scATAC-seq) of CD45^+^Lin^–^RORγt(Venus)^+^MHCII^+^ cells isolated from mLN at two weeks of age (Fig. 2a and Extended Data Fig. 3b). After quality filtering, we retained chromatin accessibility and transcriptional profiles for 10,145 cells. Unsupervised clustering of either the scRNA-seq or scATAC-seq data revealed two major cell types (Fig. 2b–d and Extended Data Fig. 3c). The first cell type represented ILC3s, spanning their full developmental spectrum including a RAG1^+^ ILC3 progenitor (ILC3p), proliferating and mature NCR^+^ ILC3s, and CCR6^+^ LTi cells (Fig. 2d and Extended Data Fig. 3c,d). The second cell type did not express canonical innate lymphoid cell (ILC) genes. This population, which was distinguished by a combination of both epithelial and dendritic cell-associated transcription factors and cell-surface molecules, consisted of four subsets and a small cluster of proliferating cells (Fig. 2b–d and Extended Data Fig. 3c–e). Although these cells expressed the dendritic cell marker *Zbtb46*, this transcript was also highly expressed by MHCII^+^ ILC3s, a finding that was confirmed by analysis of *Zbtb46*^*GFP*^*Rorgt*^*cre*^*Rosa26*^*lsl-tdTomato*^ mice (Extended Data Fig. 3f). To elucidate the identity of non-ILC3 RORγt^+^ APCs, we compared the similarity of pseudo-bulk transcriptomes across a comprehensive database of immune and stromal cells (ImmGen). As expected, ILC3 scRNA-seq clusters aligned with ILC3s, whereas the remaining clusters exhibited surprisingly high correlation with both mTECs and dendritic cells (Fig. 2e), including specific expression of AIRE, the signature mTEC transcription factor, in clusters I and III (Fig. 2f). Independent cross-referencing of these cells with immune and epithelial cell atlases using CellTypist^18^, a machine learning tool for precise cell-type annotation, also showed their overlapping transcriptional features with both dendritic cells and a generic epithelial cell (Extended Data Fig. 3g,h), consistent with their expression of p63, a critical regulator of epithelial cell differentiation^19,20^. In light of the ‘shape-shifting’ hybrid phenotype of this group of RORγt^+^ APCs, we refer to these cells as Thetis cells. Comparison of Thetis cell cluster identity defined by chromatin accessibility or gene expression revealed near perfect congruence (Extended Data Fig. 2f), confirming that Thetis cells comprise four distinct cell types (TC I–TC IV). Analysis of pseudo-bulk transcriptomes for Thetis cell sub-groups alongside published single-cell thymic epithelial transcriptomes^21^ demonstrated overlap with distinct clusters of mature mTEC subsets, in particular mature AIRE^+^ (mTEC II) and ‘post-AIRE’ (mTEC III) subsets (Fig. 2g). Overall, these data demonstrated the existence of a novel RORγt^+^ cell type present within intestinal lymph nodes during early life.

## The phenotypic landscape of Thetis cells

**Fig. 3:**
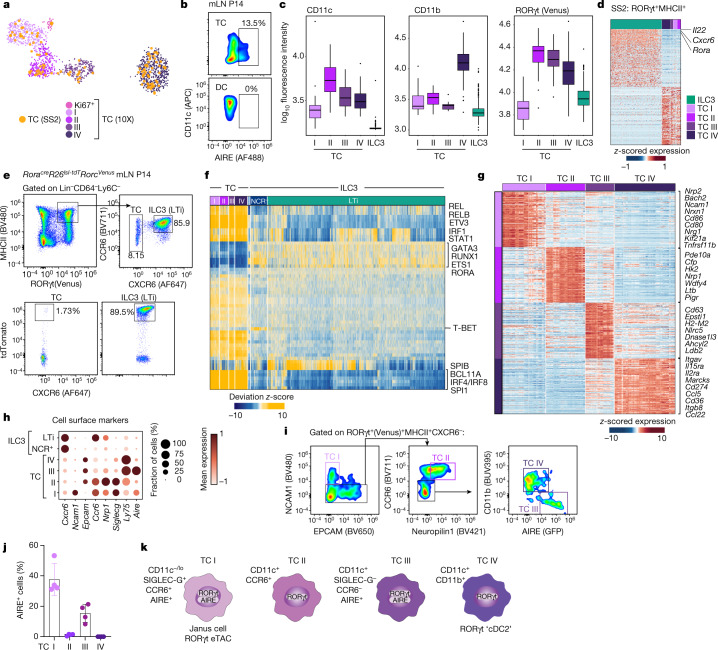
Transcriptional, epigenetic and ontological features of Thetis cell subsets. **a**, UMAP visualization of integrated 10X Genomics and Smart-seq2 (SS2) scRNA-seq analysis for RORγt^+^MHCII^+^ Thetis cells (TC), coloured by SMART-seq2 Thetis cell transcriptome or 10X cluster annotation. **b**, Intracellular expression of AIRE protein by Thetis cells and dendritic cells. **c**, Index-sorting summary graphs for CD11c, CD11b cell-surface protein and RORγt (Venus) fluorescence intensity. **d**, Heat map showing expression of top differentially expressed genes (DEGs) between Thetis cells and MHCII^+^ ILC3s, profiled by SMART-seq2, identifying *Rora* as an ILC3–Thetis cell-distinguishing gene. **e**, Representative flow cytometry analysis of tdTomato expression in MHCII^+^ILC3 and Thetis cells isolated from mLN of *Rorc*^*Venus*^*Rora*^*cre*^*Rosa26*^*lsl-tdTomato*^ fate-mapped mice at P14 (*n* = 4). **f**, Heat map reporting scaled chromVAR deviation transcription factor motif scores (left) and corresponding transcription factor gene expression values (right) for top transcription factor gene–motif pairs in Thetis cells in scATAC-seq data. **g**, Heat map showing scaled, imputed expression of top 125 DEGs (one versus the rest, fold change (FC) > 1.5, adjusted *P* < 0.01) for each Thetis cell cluster. **h**, Dot plot showing expression of selected cell-surface markers that are differentially expressed between Thetis cell subsets. **i**, Gating strategy for identification of Thetis cell subsets. **j**, Intracellular expression of AIRE protein by Thetis cell subsets; each symbol represents an individual mouse (*n* = 4). **k**, summary of TC I–TC IV phenotypes. Plots in **i** are representative of *n* = 6 mice from 3 independent experiments. Data in **b**,**e**,**j** are representative of 3 independent experiments. Box plots in **c** indicate median (centre line) and interquartile range (hinges), whiskers represent minimum and maximum values, and dots represent outliers. Source data

**Extended Data Fig. 4:**
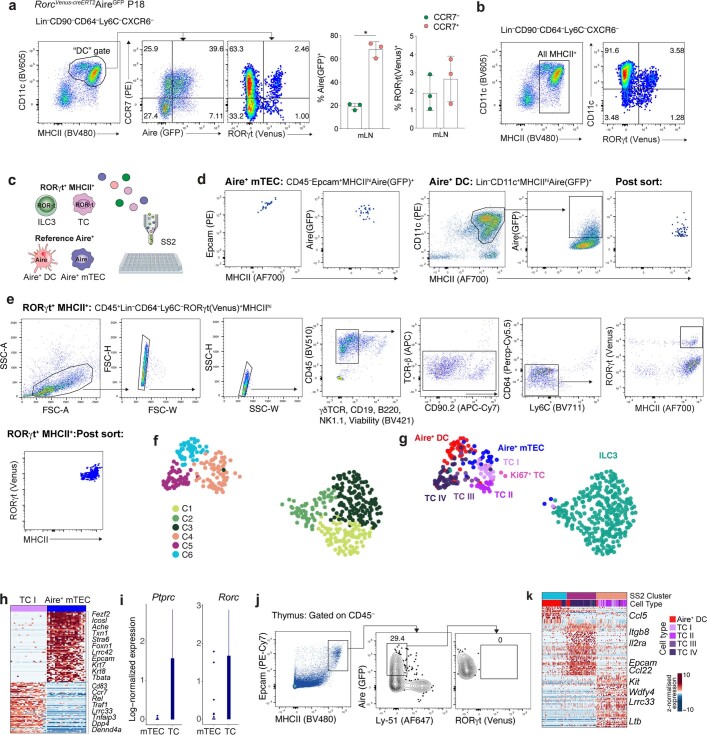
Distinguishing features of Thetis cells, mTECs and dendritic cells. **a**-**b**, Flow cytometric analysis of Lin^–^CD64^–^Ly6C^–^CD11c^+^MHCII^+^ cells (**a**) and Lin^–^ CD64^–^Ly6C^–^CXCR6^–^MHCII^+^ cells (**b**) encompassing dendritic cells (DCs) and Thetis cells (TCs) in mLN of *Rorc*^*creERT2-Venus*^Aire^GFP^ mice at P18. **c**, Schematic of single cell transcriptome profiling of RORγt^+^MHCII^+^ cells from mLN of P21 *Rorc*^*Venus-creERT2*^ mice encompassing TCs and MHCII^+^ ILC3s, alongside reference mLN Aire^+^MHCII^hi^ (CCR7^+^) DCs and thymic Aire^+^ mTECs from P21 Aire^GFP^ mice. **d**, Flow cytometry analysis of index sorted Aire^+^ mTECs or mLN Aire^+^ DCs isolated from 3-week-old Aire^GFP^ mice and (**e**) mLN Lin^–^RORγt^+^MHCII^+^ cells from 3-week-old *Rorc*^*Venus-creERT2*^ mice. **f**-**g**, UMAP visualization of 481 cells colored by (**f**) PhenoGraph cluster or (**g**) reference cell-type or RORγt^+^MHCII^+^ cell-type as assigned by mapping RORγt^+^MHCII^+^ SS2 cells to 10X scRNA-seq clusters (Fig. 2b). **h**, Heatmap reporting scaled expression values for top differentially expressed genes (FC > 1.5, adj. *P* < 0.01) between Aire^+^ mTEC and TC I. **i**, Bar graph showing log-normalized expression of *Ptprc* and *Rorc* genes in Aire^+^ mTECs and TCs. **j**, Flow cytometry of RORγt expression in Aire^+^ mTECs isolated from P18 *Rorc*^*Venus-creERT2*^Aire^GFP^ mice. **k**, Heatmap reporting scaled expression values for top differentially expressed genes (FC > 1.5, adj. *P* < 0.01) between indicated SS2 clusters (**f**). Data in **a**,**b** are representative of > 3 independent experiments. Data in **j** representative of *n* = 5 mice from two independent experiments. Source data

Extra-thymic AIRE expression has previously been reported in migratory CCR7^+^ dendritic cells^22,23^. Of note, the gene expression signature that distinguishes CCR7^+^ dendritic cells from their CCR7^–^ counterparts does not define the dendritic cell lineage, but rather represents a particular transcriptional programme that can be acquired by classical dendritic cell subsets cDC1 and cDC2, as well as other APC types^24,25^, reflecting enhanced cell migration, T cell priming capacity and expression of immune-regulatory molecules^25^. The shared expression of AIRE in Thetis cells and CCR7^+^ dendritic cells prompted us to examine the relationship between these two cell types. Analysis of *Rorc*^*Venus-creERT2*^*Aire*^GFP^ mice confirmed widespread AIRE (indicated by GFP) expression by Lin^–^CXCR6^–^CD11c^+^MHCII^+^CCR7^+^ cells encompassing both CCR7^+^ DC1 and DC2 (Extended Data Fig. 4a,b); however, less than 4% of CCR7^+^ cells expressed RORγt. Moreover, CXCR6^–^RORγt^+^MHCII^+^ Thetis cells were also found among CCR7^–^ and CD11c^−^MHCII^+^ cell populations (Extended Data Fig. 4a,b), suggesting that Thetis cells were distinct from CCR7^+^ dendritic cells. To gain further insight into the distinguishing features of AIRE-expressing Thetis cells, dendritic cells and mTECs, we performed orthogonal SMART-seq2 scRNA-seq analysis of Lin^–^RORγt^+^MHCII^+^ cells isolated from the mLN of three-week-old *Rorc*^*Venus-creERT2*^ mice in parallel with mLN AIRE^+^ dendritic cells and AIRE^+^ mTECs from age-matched *Aire*^GFP^ mice (Extended Data Fig. 4c–e). Clustering analysis, combined with mapping of SMART-seq2 transcriptomes to the droplet 10X dataset, confirmed the presence of LTi-like ILC3 and TC I–TC IV clusters (Fig. 3a and Extended Data Fig. 4f,g). Within the SMART-seq2 dataset, AIRE^+^ mTECs clustered with TC I (Extended Data Fig. 4f,g). Nevertheless, a direct comparison revealed unique expression of epithelial genes (*Foxn1*, *Krt17* and *Krt8*), the thymic marker gene *Tbata,* and *Fezf2* by mTECs, whereas TC I expressed genes associated with dendritic cells (*Ccr7*, *Cd83* and *Dpp4*) (Extended Data Fig. 4h and Supplementary Table 1). In addition, Thetis cells exclusively expressed *Ptprc* (CD45) and RORγt (Extended Data Fig. 4i), a finding confirmed by analysis of mTECs from *Rorc*^*Venus-creERT2*^ mice (Extended Data Fig. 4j). Despite overlapping markers, Thetis cells clustered separately from AIRE^+^ dendritic cells (Extended Data Fig. 4f,g), distinguished by a number of immune-regulatory genes (Extended Data Fig. 4k and Supplementary Table 2), underscoring the distinct identity of these two cell types. Furthermore, AIRE protein expression was readily detectable in 10–15% of the Thetis cell population, but not in dendritic cells (Fig. 3b), consistent with the lower level of *Aire* transcript observed in dendritic cells.

**Extended Data Fig. 5:**
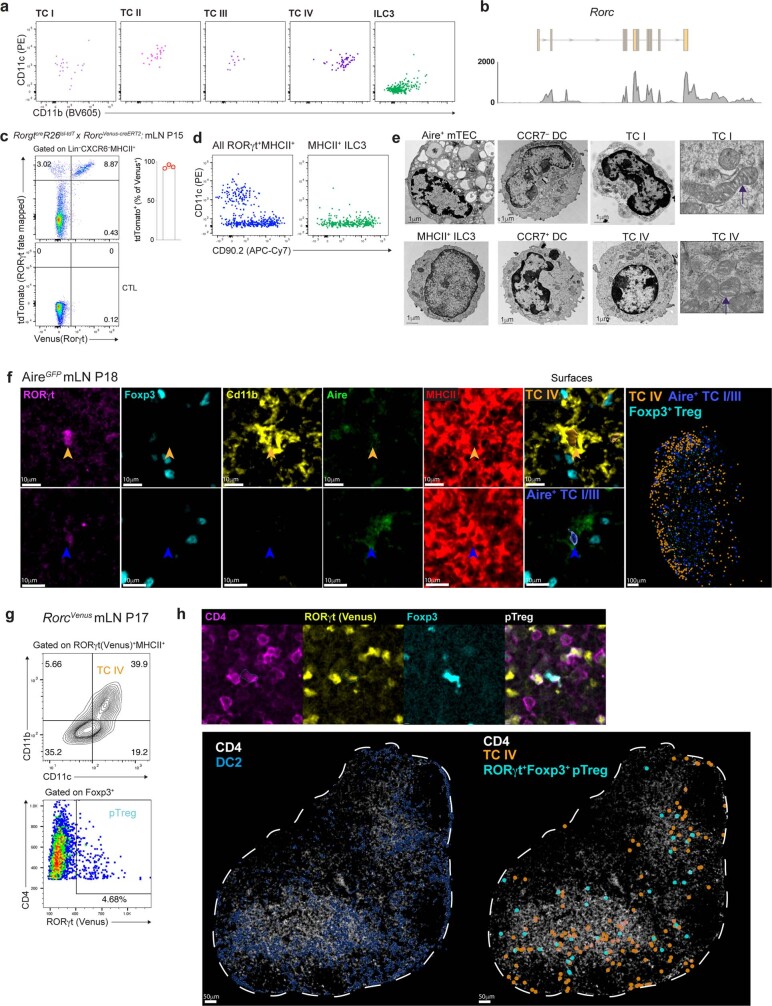
Phenotypic characterization of Thetis cells. **a**, Flow cytometry of index sorted mLN RORγt^+^MHCII^+^ cells for DC markers, CD11c and CD11b. **b**, Coverage track for smart-seq2 single cell sequencing reads mapping to the *Rorc* locus, demonstrating expression of the Rorgt isoform by TCs. **c**, Flow cytometry of Lin^–^CXCR6^–^MHCII^+^ cells from mLN of *Rorgt*^*Cre*^*R26*^*lsl-tdTomato*^*Rorc*^*Venus-CreERT2*^ mice and summary graph of frequency of tdTomato^+^ cells amongst Lin^–^CXCR6^–^Venus(YFP)^+^ cells (*n* = 3 mice). **d**, Index sorting flow cytometric analysis of all RORγt^+^MHCII^+^ cells (left panel) and cells identified as ILC3 (right panel). **e**, Electron microscopy of CCR7^–^ DCs, CCR7^+^ DCs, Aire^+^ mTECs, TC I, TC IV and MHCII^+^ ILC3 cells. Far right panel: arrows indicate distinctive mitochondrial cristae in TCs. **f**, Representative immunofluorescence imaging of TC and Treg markers in mLN sections from 2-week-old Aire^GFP^ mice. Arrowheads indicate Aire^+^ TC I/III or Cd11b^+^ TC IV. Images are representative of two independent experiments with similar results. **g**-**h**, Immunofluorescence analysis of mLN from P17 *Rorc*^*Venus*^ mice. Representative histo-cytometry plot for identification of RORγt^+^Foxp3^+^ pTreg cells and RORγt^+^MHCII^+^CD11c^+^CD11b^+^ TC IV (**g**) and representative immunofluorescence imaging demonstrating distribution of indicated cell types (**h**). *n* = 3 mice, 2 lymph nodes per mouse. Data in **c** are representative of 2 independent experiments. Source data

Index-sorting analysis of cell-surface markers revealed that Thetis cells spanned a spectrum from CD11c^–/lo^ (TC I) to CD11c^hi^ (TC II–TC IV) cells (Fig. 3c and Extended Data Fig. 5a). In addition, TC IV was distinguished by high levels of CD11b expression. These findings suggest that RORγt^+^CD11c^+^CD11b^+^MHCII^+^ cells, previously identified among Tbet^–^ cDC2B^15^, represent TC IV. TC II–TC IV expressed higher levels of RORγt (indicated by Venus) than ILC3s (Fig. 3c), reflecting *Rorgt* promoter activity (Extended Data Fig. 5b). Although TC I expressed lower levels of RORγt, all Thetis cell subsets expressed sufficient levels of *Rorgt* to drive *Rorgt*-cre-mediated recombination, as evidenced by the near universal expression of tdTomato by CXCR6^–^Venus(RORγt)^+^MHCII^+^ Thetis cells isolated from *Rorc*^*Venus-creERT2*^*Rorgt*^*cre*^*Rosa26*^*lsl-tdTomato*^ mice (Extended Data Fig. 5c). Of note, ILC3s, traditionally identified as CD90^+^ cells, encompassed both CD90^–^ and CD90^+^ cells and did not express CD11c (Fig. 3c and Extended Data Fig. 5d). To assess whether the partially overlapping transcriptional features of Thetis cells with dendritic cells and mTECs were coupled to similar or distinct morphological attributes, we analysed Thetis cells by electron microscopy. Dendritic cells, AIRE^+^ mTECs and MHCII^+^ ILC3 cells served as reference populations. Although CD11c^–^RORγt^+^AIRE^+^ cells have previously been described as lymphoid^16^, electron microscopy analysis revealed that both CD11c^–^AIRE^+^ TC I and CD11c^+^ Thetis cells more closely resembled myeloid cells, in contrast to MHCII^+^ ILC3s, which had a typical lymphoid appearance (Extended Data Fig. 5e). Thetis cells were distinct from classical CCR7^+^ and CCR7^–^ dendritic cells as well as mTECs, and featured distinctive mitochondria with rounded, condensed cristae. To further probe Thetis cell localization and morphology, we examined their spatial distribution in the mLN of *Aire*^*GFP*^ mice at P18. Immunofluorescence staining confirmed the presence of AIRE^+^ Thetis cells (TC I and TC III) as well as AIRE^–^CD11b^+^ TC IV, with TC IV preferentially located within the paracortex (Extended Data Fig. 5f). Analysis of mLN from *Rorc*^*Venus-creERT2*^ mice confirmed similar spatial distributions of TC IV and RORγt^+^ pT_reg_ cells (Extended Data Fig. 5g,h).

## Thetis cell ontogeny

**Extended Data Fig. 6:**
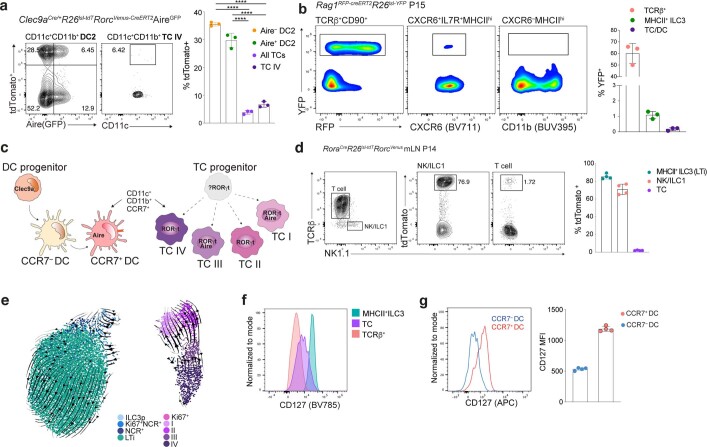
Thetis cells are ontogenically distinct from dendritic cells and ILC3s. **a**, tdTomato labeling in cDC and TC from mLN of DC fate-mapping RORγt and Aire double reporter (*Clec9a*^*Cre/+*^*R26*^*lsl-tdTomato*^*Rorc*^*Venus-creERT2*^Aire^GFP^) mice at P18. **b**, Flow cytometry analysis of TCRβ^+^, MHCII^+^ ILC3, and CXCR6^–^MHCII^+^ cells encompassing TCs and DCs, from mLN of RAG1 fate-mapped (*Rag1*^*creERT2*^*R26*^*lsl-YFP*^) mice (*n* = 3) at P15 following 4-OHT treatment on P3, 5 and 7. **c**, Schematic of DC and TC ontogeny demonstrating distinct and overlapping transcriptional regulators and cell surface markers. **d**, Flow cytometry analysis of indicated immune cell subsets from mLN of RORα fate-mapped *Rorc*^*Venus*^ mice and summary bar graph for tdTomato labeling. **e**, UMAP of RORγt^+^MHCII^+^ cells (Fig. 2b) with scVelo-projected velocities, shown as streamlines. **f**, IL7R (CD127) expression (representative of *n* = 4 mice) on ILC3s and TCs. **g**, Expression of IL7R by DC subsets. Each dot represents an individual mouse, (*n* = 4). Data in **a**,**b**,**d**,**f**,**g** are representative of 2-3 independent experiments. Source data

To address the ontogeny of Thetis cells, we first analysed dendritic cell fate-mapping *Clec9a*^*cre/wt*^*Rosa26*^*lsl-tdTomato*^*Rorc*^*Venus-creERT2*^*Aire*^*GFP*^ reporter mice in which both cDC1 and cDC2 are labelled owing to CLEC9A expression in dendritic cell progenitors^26^. In contrast to both AIRE^+^ and AIRE^–^ DC2s, less than 5% of Thetis cells were tdTomato^+^ (Extended Data Fig. 6a), probably reflecting the small proportion of Thetis cells with detectable *Clec9a* expression (Extended Data Fig. 3e), rather than descendancy from a CLEC9A^+^ pre-dendritic cell. Given reports of a lymphoid pathway for cDC differentiation^27^, we next used a novel *Rag1*^*RFP-creERT2*^*Rosa26*^*lsl*-YFP^ mouse to fate-map progeny of lymphoid progenitor cells following neonatal administration of 4-hydroxytamoxifen (4-OHT). In contrast to labelling of T cells and ILC3s, YFP^+^ cells were absent among Lin^–^MHCII^hi^CXCR6^–^ cells encompassing Thetis cells (Extended Data Fig. 6b). These findings suggested that Thetis cells are ontogenically and transcriptionally distinct from classical dendritic cells. Thus, the overlapping phenotype of Thetis cells and dendritic cells, in particular CCR7^+^ dendritic cells, probably reflects shared functions related to cell migration and antigen presentation rather than shared ontogeny (Extended Data Fig. 6c). RORγt^+^AIRE^+^ cells have previously been suggested to be related to ILC3s^16,28^, however, the absence of *Rag1* fate-mapped Thetis cells suggested that Thetis cells were not descended from the RAG1^+^ ILC3p identified within our scRNA-seq dataset (Extended Data Fig. 3c). Furthermore, Thetis cells did not express RORα (Fig. 3d), a critical gene for ILC development that is expressed by ILC precursors (ILCp) and mature ILC subsets^29–31^. The exclusive expression of RORα by MHCII^+^ ILC3s enabled us to determine the lineage relationship between ILCp and Thetis cells using *Rorc*^*Venus*^*Rora*^*cre*^*Rosa26*^*lsl-tdomato*^ fate-mapping mice. Within the mLN, around 90% of LTi cells and 70% of ILC1 and natural killer cells were tagged with tdTomato, compared with less than 2% of Thetis cells (Fig. 3e and Extended Data Fig. 6d), confirming that Thetis cells are not developmentally related to ILCs. To address the possibility of the reverse relationship—that Thetis cells are the precursor to ILC3s—we analysed single-cell transcriptional dynamics using single-cell RNA velocity^32^ to computationally define lineage relationships between Thetis cells and ILC3s. This analysis identified established differentiation trajectories emanating from ILC3p to NCR^+^ ILC3s and LTi cells (Extended Data Fig. 6e). In contrast, no ‘connectivity’ was observed between Thetis cells and ILC3s in either direction. Although IL7R is expressed by ILCs and a subset of Thetis cells (Extended Data Fig. 6f), it is also expressed by CCR7^+^ dendritic cells^23^ (Extended Data Fig. 6g), consistent with recently reported IL7R-cre fate-mapping in around 25% of cDCs^33^, precluding this approach for lineage tracing ILCs, Thetis cells and dendritic cells. Together these results demonstrate that Thetis cells represent a new class of cells, distinct from both ILCs and classical dendritic cells.

To determine the transcription factors that regulate Thetis cell differentiation and heterogeneity, we turned to our scATAC-seq data, integrating differential transcription factor motif activity with gene expression. This analysis identified activity of canonical ILC3 transcription factors in ILC3s, including RORα, GATA3 and TCF1, as well T-BET (encoded by *Tbx21*) in NCR^+^ ILC3s (Fig. 3f), validating our approach. By contrast, Thetis cells were distinguished by activity of a unique group of transcription factors including Spi-B, a critical regulator of mTEC differentiation, as well as core transcription factors governing myeloid cell differentiation (PU.1 (encoded by *Spi1*), BCL11A, IRF8 and IRF4) (Fig. 3f), in agreement with their transcriptional overlap with both mTECs and dendritic cells. Notably, several of the signature Thetis cell transcription factors have been shown to regulate AIRE expression in mTECs^34^, suggesting a common transcriptional network between these two cell types. Together, these findings establish the unique identity of Thetis cells, delineating their shared and distinct features with both mTECs and dendritic cells.

## Transcriptional programmes of Thetis cell subsets

**Extended Data Fig. 7:**
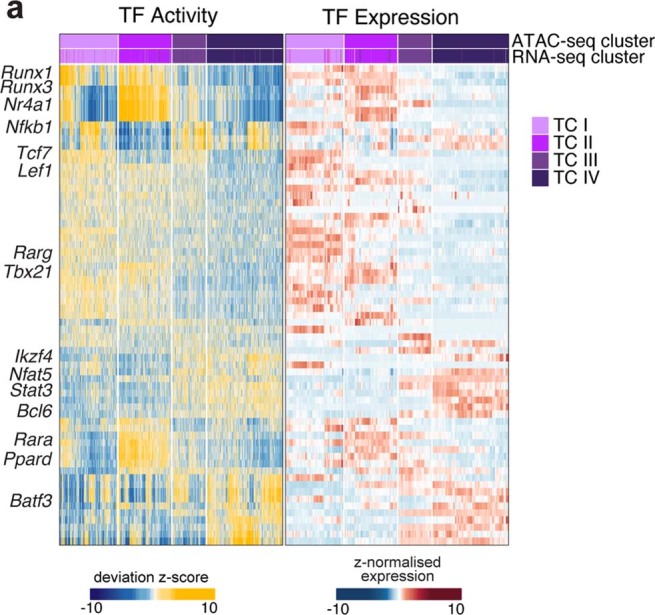
Characterization of Thetis cell subsets. **a**, Heatmap reporting transcription factor (TF) motif activity score (left panel) or TF gene expression (right panel) for top TF-motif and gene expression pairs in scATAC/RNA-seq data (Fig. 1b).

To gain insight into the potential functions of Thetis cell subsets, we examined their distinguishing transcriptional features (Fig. 3g). TC I expressed canonical AIRE^+^ mTEC genes, including *Aire*, *Cd80*, *Cd86*, *Tnfrsf11b* (which encodes OPG), and genes associated with neuronal adhesion, signalling and growth (*Nrxn1*, *Nrn1* and *Ncam1*). Of note, a recent study of peripheral AIRE-expressing cells identified a population of ‘mTEC-like’ RORγt^+^AIRE^+^ cells within lymph nodes, similarly distinguished by neuronal genes^17^, probably representing AIRE^+^ TC I. TC II was distinguished by exclusive expression of several distinctive genes including *Pigr* and *Cldn7*, signature molecules for a group of mTECs with a history of AIRE expression^21,35^, further highlighting parallels between the mTEC and Thetis cell subsets. TC III expressed high levels of AIRE as well as *Nlrc5*, a critical regulator of MHC Class I genes. TC IV expressed immune-regulatory genes (*Cd274*) as well as genes associated with cell migration (*Marcks* and *Cxcl16)*. Analysis of differential chromatin accessibility and motif enrichment across Thetis cell subsets suggested several subset-specific transcriptional regulators, further underpinning the observed heterogeneity of Thetis cells (Extended Data Fig. 7a).

To validate the observed Thetis cell phenotypes, we devised a panel of flow cytometry markers (Fig. 3h). MHCII^+^ ILC3s were distinguished from Thetis cells by the expression of CXCR6 (Fig. 3d,e,h). Among CXCR6^–^RORγt^+^MHCII^hi^ cells, we confirmed the presence of Thetis cell subsets expressing signature cell-surface markers (Fig. 3i) with the expected pattern of AIRE protein expression in TC I and TC III (Fig. 3j). Of note, CCR6 and SIGLEC-G, which have been suggested to be markers for RORγt^+^AIRE^+^ cells^16,36,37^﻿, were expressed by TC I and TC II but not by TC III and TC IV (Fig. 3h,i). Thus, whereas AIRE^+^ TC I represent RORγt^+^AIRE^+^cells, referred to either as Janus cells or RORγt^+^ extra-thymic AIRE-expressing cells (eTACs) in two concurrent analyses of RORγt^+^ APCs^36,37^, TC II–TC IV extend the spectrum of non-ILC3 RORγt^+^MHCII^+^ cells beyond AIRE-expressing cells (Fig. 3k). Together, our analyses suggest that Thetis cell subsets are molecularly and functionally distinct and point to a role for TC IV in pT_reg_ differentiation.

## ILC3 and DCs are dispensable for pT_reg_ generation

**Fig. 4:**
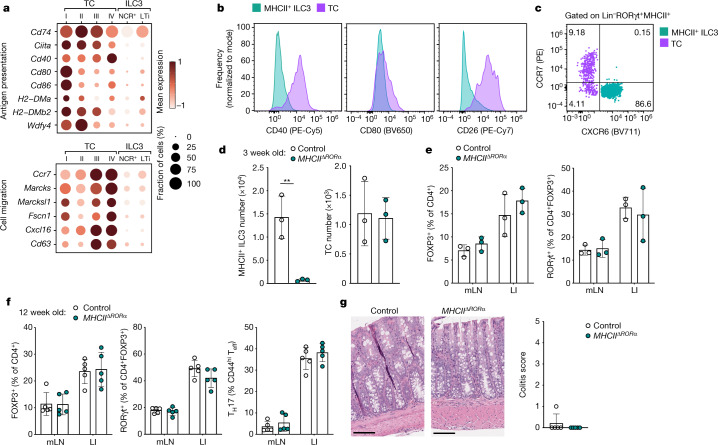
Antigen presentation by ILC3s is not required for intestinal pT_reg_ differentiation. **a**, Dot plot showing expression of genes related to antigen presentation, T cell priming and cell migration across Thetis cell and MHCII^+^ ILC3 clusters (Fig. 2b). **b**, Representative flow cytometry of mLN ILC3s (CXCR6^+^RORγt^+^MHCII^+^) and Thetis cells (CXCR6^–^RORγt^+^MHCII^+^) from P18 *Rorc*^*Venus-creERT2*^*Aire*^*GFP*^ mice (*n* = 3), showing expression of indicated chemokine receptors, co-stimulatory and immune-regulatory molecules. **d**,**e**, Immune cell composition of 3-week-old *MHCII*^*ΔRORα*^ (*n* = 3) and *Rora*^cre^*H2-Ab1*^*fl/wt*^ (*n* = 3) mice. **d**, Number of MHCII^+^ ILC3s and Thetis cells in mLN. **e**, Frequency of total T_reg_ (FOXP3^+^) and RORγt^+^ pT_reg_ cells. **f**, Frequency of total T_reg_ (FOXP3^+^), RORγt^+^ pT_reg_ cells and T_H_17 cells in mLN and large intestine lamina propria (LI) of 12-week-old *MHCII*^*ΔRORα*^ (*n* = 5) and *Rora*^cre^*H2-Ab1*^*fl/wt*^ (*n* = 5) mice. **g**, Representative histological analysis of H&E-stained sections of the colon of mice in **f** (left) and summary histological colitis score (right). Scale bars, 200 μm. Data in **b**–**f** are representative of two or three independent experiments. Data are mean ± s.e.m. Each symbol represents an individual mouse. Two-tailed unpaired *t*-test. Source data

**Extended Data Fig. 8:**
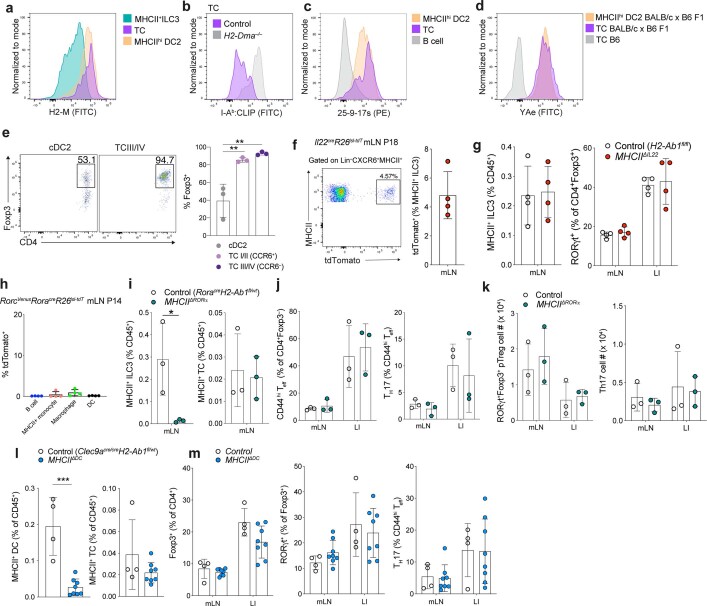
Antigen presentation by ILC3s or dendritic cells is not required for extra-thymic intestinal pTreg differentiation. **a**–**d**, Flow cytometry of mLN from P14 *Rorc*^*Venus-creERT2*^ (**a**,**c**) or *H2-Dma*^*–/–*^ and littermate wild-type mice (**b**) or BALB/c x B6 F1 *Rorc*^*Venus-creERT2*^ mice (**d**). demonstrating expression of indicated antigen processing and presenting molecules. MHCII^+^ ILC3s: (Lin^–^CXCR6^+^RORγt^+^MHCII^+^, TCs: (Lin^–^CXCR6^–^RORγt^+^MHCII^+^ and DC2s: (Lin^–^RORγt^–^CD11c^+^MHCII^hi^CD11b^+^. Representative of *n* = 4 mice. **e**, frequency of Foxp3^+^ T cells amongst CD4^+^ T cells following co-culture of naïve CD4^+^ C7 T cells with indicated TC or DC subset. **f**, tdTomato labeling in MHCII^+^ILC3 (Lin^–^CXCR6^+^MHCII^+^) from mLN of IL22 fate-mapping (*Il22*^*Cre/+*^*R26*^*lsl-tdtomato*^) mice at P18. Representative flow cytometry and summary bar graph, n = 4 mice. **g**, Frequency of MHCII^+^ ILC3s and RORγt^+^ pTreg amongst CD4^+^Foxp3^+^ cells in indicated tissues from 3-week-old *MHCII*^*ΔIL22*^ (*n* = 4) and control (*H2-Ab1*^*fl/fl*^) (*n* = 4) mice. **h**, tdTomato expression by MHCII^+^ cell types in mLN of *Rorc*^*Venus*^*Rora*^*cre*^*R26*^*lsl-tdTomato*^ fate-mapped mice at P14*; n* = 4 mice. **i**-**j**, Immune cell composition of 3-week-old *MHCII*^*ΔRORα*^ (*n* = 3) and *Rora*^cre^*H2-Ab1*^*fl/wt*^ (*n* = 3) mice. (**i**) Frequency of MHCII^+^ ILC3s and TCs in mLN. (**j**), Frequency of CD4^+^Foxp3^–^CD44^hi^ T_eff_ and RORγt^+^ Th17 cells, (**k**) Total number of RORγt^+^Foxp3^+^ pTreg and RORγt^+^ Th17 cells. **l**-**m**, Immune cell composition in mLN of 3-week-old *MHCII*^*ΔDC*^ (*n* = 4) and control (*Clec9a*^cre^*H2-Ab1*^*fl/wt*^) (*n* = 8) mice from 2 independent experiments. Frequency of MHCII expressing DCs or TCs within mLN (**l**). Frequency of total Foxp3^+^ Treg cells, pTreg cells amongst CD4^+^Foxp3^+^ cells, and Th17 cells in mLN and LI (**m**). Data in **a**-**k** are representative of 2-3 independent experiments, data in **l**,**m** are pooled from 2 independent experiments. Error bars: means ± s.e.m. ***P < 0.01, ****P < 0.0001; unpaired two-sided *t*-test. Source data

Given the overlapping phenotype of Thetis cells with professional APC types with known roles in T cell tolerance, we hypothesized that Thetis cells were the relevant RORγt^+^MHCII^+^ cell type for instructing pT_reg_ cell differentiation. A direct comparison of Thetis cell and ILC3 transcriptomes, as well as cell-surface protein expression, confirmed that Thetis cells were enriched for molecules associated with antigen presentation, T cell activation and cell migration, in contrast to MHCII^+^ ILC3 cells (Fig. 4a,b and Extended Data Fig. 8a). Furthermore, in contrast to Thetis cells, we did not observe CCR7 protein expression on MHCII^+^ ILC3s (Fig. 4c), despite detectable *Ccr7* transcript. To examine the antigen-presenting ability of Thetis cells, we analysed cell-surface I-A^b^ bound CLIP peptide on *H2-Dma*^–/–^ or littermate wild-type Thetis cells, which confirmed efficient H-2M-mediated CLIP displacement (Extended Data Fig. 8b). Accordingly, staining with the 25-9-17s monoclonal antibody, which binds to a subset of non-CLIP peptide–I-A^b^ complexes^38^, demonstrated equivalent levels of expression by Thetis cells and classical dendritic cells (Extended Data Fig. 8c). To further examine MHC class II antigen presentation by Thetis cells, we bred *Rorc*^*Venus-creERT2*^ with BALB/c mice and confirmed expression of an endogenously processed self-peptide Eα52-68 bound I-A^b^, using the YAe monoclonal antibody (Extended Data Fig. 8d). To address the ability of Thetis cells to induce pT_reg_ cells *ex vivo*, we co-cultured Thetis cell subsets (either CCR6^+^ TC I and TC II or CCR6^–^ TC III and TC IV) with naive C7 TCR transgenic CD4^+^ T cells and their cognate peptide under suboptimal T_reg_-inducing conditions. Notably, Thetis cells demonstrated significantly greater ability to promote T_reg_ differentiation relative to cDC2s with the greatest efficacy observed among TC III and TC IV (92.7% FOXP3^+^ for TC III and TC IV versus 39.4% for cDC2; Extended Data Fig. 8e). Together, these results suggest that Thetis cells are competent APCs.

Our earlier analysis identified exclusive expression of IL-22 by ILC3s (Fig. 3d), suggesting the potential utility of *MHCII*^*ΔIl22*^ mice to determine the role of antigen presentation by ILC3s in pT_reg_ induction. However, consistent with the low frequency of fate-mapped MHCII^+^ILC3s in *Il22*^*cre*^*Rosa*^*lsl-tdTomato*^ mice (less than 5%; Extended Data Fig. 8f), *MHCII*^*ΔIl22*^ mice exhibited only minimal loss of MHCII expression by ILC3s with no effect on the pT_reg_ cell population (Extended Data Fig. 8g). We therefore turned to *Rora*^cre^ mice as a means to selectively target ILC3s but not Thetis cells. Notably, ILC3s were the only MHCII^+^ RORα fate-mapped cell type within the mLN (Extended Data Figs. 6d and 8h), establishing *MHCII*^*ΔRORα*^ mice as a genetic model for studying the functional role of ILC3 antigen presentation. Indeed, analysis of three-week-old *MHCII*^*ΔRORα*^ mice confirmed a complete loss of MHCII expression on ILC3s, with no changes in Thetis cells (Fig. 4d and Extended Data Fig. 8i). Conspicuously, the intestinal T cell composition was not perturbed in *MHCII*^*ΔRORα*^ mice, with equivalent proportions and numbers of CD4^+^ T_eff_ and T_reg_ cells, including RORγt^+^ pT_reg_ cells, within the mLN and large intestine (Fig. 4e and Extended Data Fig. 8j,k). To exclude a role for ILC3s in pT_reg_ differentiation in later life, we examined *MHCII*^*ΔRORα*^ mice at 12 weeks of age. In contrast to *MHCII*^*ΔRORgt*^ mice, *MHCII*^*ΔRORα*^ adult mice had normal pT_reg_ frequencies, with no evidence of altered T cell activation states (Fig. 4f), and lacked histological signs of colonic inflammation (Fig. 4g), further confirming that MHCII-mediated antigen presentation by ILC3s is not required for intestinal tolerance. Besides ILC3s, antigen presentation by sub-immunogenic dendritic cells is thought to favour T cell tolerance. Although Thetis cells may have been inadvertently targeted by studies using ‘dendritic cell-specific’ Cre drivers^39^ owing to expression of both CD11c and Zbtb46, the absence of Clec9a fate-mapped Thetis cells enabled us to revisit a role for classical dendritic cell in pT_reg_ differentiation through analysis of *Clec9a*^*cre/cre*^*H2-Ab1*^*fl/fl*^ (*MHCII*^*ΔDC*^) mice in which dendritic cells, but not Thetis cells, were rendered MHCII-deficient (Extended Data Fig. 8l). Notably, we did not observe changes in RORγt^+^FOXP3^+^ cells in these mice (Extended Data Fig. 8m). Overall, these findings demonstrate that MHCII antigen presentation by ILC3s or cDCs is dispensable for pT_reg_ cell differentiation, leaving Thetis cells as the pT_reg_-inducing RORγt^+^ APC.

## Thetis cells induce pT_reg_ cells in early life

**Fig. 5:**
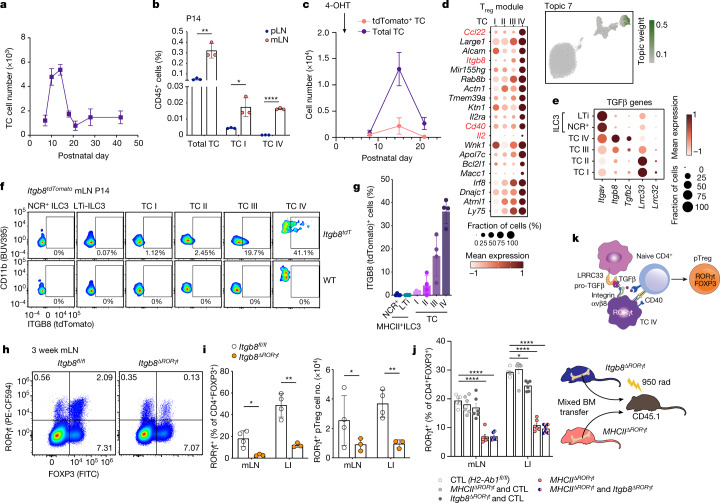
A developmental wave of Thetis cells promotes early life pT_reg_ differentiation in an ITGB8-dependent manner. **a**, Number of Thetis cells in mLN from P7 to week 6 (*n* = 3–8 individual mice per timepoint). **b**, Frequency of Thetis cells in pLN and mLN of *Rorc*^*Venus-creERT2*^*Aire*^*GFP*^ mice at P14 (*n* = 3 mice per group). **c**, Total number of tdTomato^–^ and tdTomato^+^ Thetis cells isolated from mLN of *Rorc*^*Venus-creERT2*^*Rosa26*^*lsl-tdTomato*^*Aire*^*GFP*^ mice at indicated time intervals following administration of 4-OHT at P1 (*n* = 4 mice per timepoint). **d**, Topic modelling of 10X scRNA-seq Thetis cell transcriptomes. The UMAP is coloured by the weight of topic 7 in each cell. **e**, Dot plot showing the expression of TGFβ pathway genes in Thetis cells and ILC3s. **f**,**g**, Representative flow cytometry (**f**) and summary graphs (**g**) of ITGB8 (tdTomato) expression in Thetis cell and ILC3 subsets in mLN of *Itgb8*^*tdTomato*^ (*n* = 4) or littermate wild-type (WT) mice. **h**,**i**, Representative flow cytometry of RORγt- and FOXP3-expressing T cell subsets (**h**) and summary graphs for frequencies and numbers (**i**) of pT_reg_ (RORγt^+^FOXP3^+^) cells in mLN and large intestine lamina propria (LI) of 3-week-old *Itgb8*^*ΔRORγt*^ (*n* = 3) and *Itgb8*^*fl/fl*^ (*n* = 4) mice. **j**, Frequency of RORγt^+^ pT_reg_ cells among CD4^+^FOXP3^+^ cells in mLN and large intestine lamina propria of mixed bone marrow (BM) chimeras, analysed 6 weeks after reconstitution (*n* = 6 mice per group). **k**, Schematic of pT_reg_ induction by Thetis cells. Data in **b**,**c** are representative of two independent experiments. Data in **f**,**g**,**j** are pooled from two (**j**) or three (**f**,**g**) independent experiments. Data in **h**,**i** are representative of 4 independent experiments. Data are mean ± s.e.m. Two-tailed unpaired *t*-test. Source data

**Extended Data Fig. 9:**
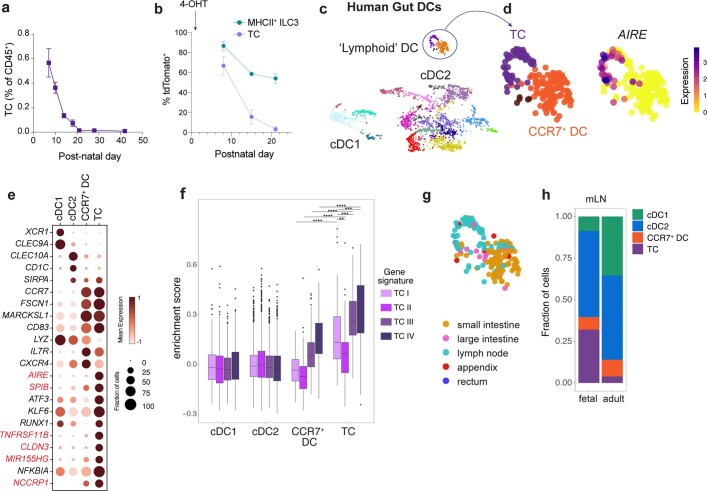
Thetis cells are enriched in early life and conserved across mouse and human. **a**, Frequency of TCs within mLN from postnatal day 7 to 6-weeks-of age (*n* = 3–8 mice per timepoint). **b**, Percentage of tdTomato^+^ TCs and MHCII^+^ ILC3s isolated from mLN of *Rorc*^*Venus-creERT2*^*R26*^*lsl-tdTomato*^Aire^GFP^ mice at indicated time intervals following administration of 4-OHT on P1 (*n* = 4 mice per timepoint). **c**, Human gut atlas single cell transcriptomes. Cells annotated as DCs were reclustered with PhenoGraph and visualized with UMAP. **d**, UMAP of ‘lymphoid’ DC clusters colored by PhenoGraph cluster or unimputed expression of AIRE. **e**, Dot plot showing select genes differentially expressed between indicated cell subsets. **f**, Enrichment of TC subset signature genes within indicated human APC subsets. **g**, UMAP colored by tissue of origin. **h**, Proportion of indicated DC/TC subsets within mLN samples in fetal *vs* adult samples. Clusters annotated as cDC2 or cDC1 were grouped for analysis. Data in **b** are representative of two independent experiments. Box plots (**f**) indicate the median (center lines) and interquartile range (hinges), and whiskers represent min and max, dots represent outliers. ****P < 0.0001, ***P < 0.001, **P < 0.01; Mann Whitney U test (**f**). Source data

Given the narrow temporal window of opportunity for establishing intestinal immune tolerance, we hypothesized that the presence of Thetis cells might determine this developmental window. Our analysis of Thetis cell abundance in mice ranging in age from 7 days to 6 weeks revealed their marked enrichment between 1 and 3 weeks of age, with a rapid decline thereafter (Fig. 5a and Extended Data Fig. 9a). Notably, Thetis cells—in particular TC IV—were enriched in the mLN compared with skin-draining peripheral lymph nodes (pLN) (Fig. 5b). To determine the dynamics of neonatal Thetis cell differentiation, we used *Rorc*^*Venus-creERT2*^*Rosa26*^*lsl-tdTomato*^ mice to label RORγt-expressing cells and their progeny. Following treatment of mice with 4-OHT at P1, more than 60% of Thetis cells remained tdTomato^+^ at P7 (Extended Data Fig. 9b). This proportion fell to 15% by P14, although total numbers of both tdTomato^–^ and tdTomato^+^ Thetis cells increased between P7–P14 (Fig. 5c), reflecting de novo Thetis cell differentiation or proliferation during this critical developmental window. Both the proportion of tdTomato^+^ Thetis cells and total cell numbers declined from P14, reflecting waning differentiation beyond this age (Fig. 5c and Extended Data Fig. 9b). By contrast, the proportion of fate-mapped MHCII^+^ ILC3s declined between P7 and 14 but remained stable thereafter (Extended Data Fig. 9b), consistent with the notion that ILC3s are maintained by self-renewal^40^. Thus, Thetis cells or putative RORγt^+^ Thetis cell progenitors are present at birth, and are prominently enriched within the mLN at the time of induction of intestinal tolerance during early life. Together these data suggest a critical window of opportunity for pT_reg_ cell differentiation, determined by a wave of Thetis cells in the mLN in early life.

To determine whether Thetis cell counterparts exist in humans, we analysed a recent single-cell atlas encompassing second trimester to adult intestine and mLN^41^. Within a group of myeloid cells annotated as ‘lymphoid’ dendritic cells, we identified a cluster of cells distinct from but closely related to CCR7^+^ dendritic cells, that expressed signature Thetis cell genes (*TNFRSF11B* and *SPIB*), including high levels of *AIRE* (Extended Data Fig. 9c–e). Analysis of orthologous signature Thetis cell subset genes confirmed enrichment in the putative human Thetis cell cluster, most prominently for TC III- and TC IV-defining genes (Extended Data Fig. 9f). In contrast to CCR7^+^ dendritic cells, human Thetis cells were present almost exclusively in the mLN (Extended Data Fig. 9g) and were highly enriched in fetal samples (32% versus 3.8%; Extended Data Fig. 9h), implying a conserved role for Thetis cells in intestinal tolerance during early life.

**Extended Data Fig. 10:**
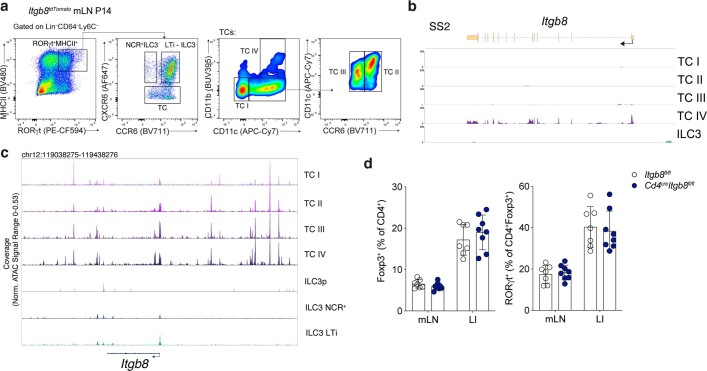
Thetis cells promote intestinal pTreg differentiation in an Itgb8-dependent manner. **a**, Gating strategy for identification of ILC3 and TC subsets in mLN of *Itgb8*^*tdTomato*^ mice at P14 (representative of *n* = 4 mice, three independent experiments). **b**, *Itgb8* transcript levels in TC and ILC3 subsets profiled by Smart-seq 2. **c**, Chromatin accessibility at the *Itgb8* locus and Itgb8 transcript levels in TC and ILC3 subsets (cells as in Fig 1c). **d**. Frequency of total Foxp3^+^ Treg cells and percentage of RORγt^+^ pTreg cells in mLN and LI of *Itgb8*^*ΔCd4*^ (*n* = 8) or Itgb8^fl/fl^ mice (*n* = 7). Data pooled from two (**d**) or 3 (**a**) independent experiments. Error bars: means ± s.e.m. NS, not significant; unpaired two-sided *t*-test. Source data

The close temporal and spatial relationship between TC IV and pT_reg_ cells supported a key role for this Thetis cell subset in pT_reg_ differentiation. To determine whether the TC IV subset has distinct features supporting pT_reg_ generation besides MHCII-dependent TCR stimulation, we used latent Dirichlet allocation—a probabilistic topic model—to capture shared and unique gene expression programmes. Notably, the TC IV subset was enriched for a ‘T_reg_-inducing’ module encompassing critical molecules including IL-2, the TGFβ-activating integrin ITGΒ8, CD40 and CCL22 (Fig. 5d). TGFβ signalling is known to be critical for pT_reg_ cell differentiation. Activation of latent extracellular TGFβ requires physical interaction with integrin αvβ6 or integrin αvβ8, and loss of either TGFβ signalling on T cells, or ITGΒ8 expression by hematopoietic cells, leads to impaired pT_reg_ differentiation and development of autoimmunity and colitis^42,43^. Analysis of TGFβ signalling pathway genes in Thetis cell transcriptomes confirmed high expression of both *Itgav* and *Itgb8* in TC IV, as well as unique expression of *Tgfb2* (Fig. 5e). To address the role of Thetis cells in TGFβ-mediated pT_reg_ differentiation, we generated mice with conditional loss of the *Itgb8* gene in RORγt^+^ cells (*Itgb8*^*ΔRORγt*^). Although ILC3s expressed Itgav, they did not express *Itgb8*, as determined by analysis of SMART-seq2 single-cell transcriptomes as well as *Itgb8*^*tdTomato*^ reporter mice (Fig. 5f,g and Extended Data Fig. 10a,b). Furthermore, scATAC-seq analysis showed that the *Itgb8* locus was inaccessible in ILC3s (Extended Data Fig. 10c), confirming that *Itgb8*^*ΔRORγt*^ mice have a specific deficiency of ITGB8 in Thetis cells and T cells. Analysis of mLN and large intestine from three-week-old mice, revealed a substantial reduction in pT_reg_ cell frequency and numbers (Fig. 5h,i), mirroring the loss of pT_reg_ cells observed in *MHCII*^*ΔRORγt*^ mice. Differentiation of pT_reg_ cells was normal in *Cd4*^*cre*^*Itgb8*^*fl/fl*^ mice (Extended Data Fig. 10d), indicating that TGFβ activation by Thetis cells—and not by T cells—is a critical mechanism for intestinal pT_reg_ cell differentiation.

The unique expression of *Itgb8* by Thetis cells enabled us to test the requirement for TGFβ activation and antigen presentation by the same cell. Using bone marrow chimeras generated with a mix of *Itgb8*^*ΔRORγt*^ and *MHCII*^*ΔRORγt*^ bone marrow cells—in which both ILC3s and Thetis cells can present antigen, but the same Thetis cell cannot present antigen and activate TGFβ—we found that pT_reg_ differentiation was critically dependent on antigen presentation by *Itgb8*-expressing Thetis cells, with an equivalent deficit in pT_reg_ cells observed in *MHCII*^*ΔRORγt*^ and *MHCII*^*ΔRORγt*^*/Itgb8*^*ΔRORγt*^ chimeras (Fig. 5j), excluding the possibility of redundant and compensatory functions between Thetis cells and ILC3s. Overall, our findings identify a new class of APCs that is prominent in the mLN during a critical early life window, and demonstrate an essential role for the TC IV subset in establishing intestinal tolerance through the generation of pT_reg_ cells (Fig. 5k).

## Discussion

Contrary to the view that ‘neonatal immune privilege’—first demonstrated by Medawar in the 1950s^44,45^—results from the presence of immunosuppressive or ‘immature’ dendritic cells with an inferior stimulatory capacity, our results suggest the existence of a dedicated tolerogenic APC type that is enriched in early life. The requirement for MHC class II antigen presentation by Thetis cells in early life but not in adulthood provides support for a model in which a tightly regulated wave of Thetis cell differentiation during a narrow developmental window imprints durable microbiota-specific T cell tolerance. Of note, pT_reg_ cell abundance in adulthood is determined by cues sensed during the first week of life^8^, coincident with the observed wave of Thetis cell differentiation, suggesting that modulation of Thetis cell development may have lasting effects on intestinal immune tolerance.

A defining feature of Thetis cells is their expression of RORγt. Our finding of overlapping markers between Thetis cells and dendritic cells resolves previous conflicting reports on RORγt^+^ dendritic cells and their relationship to ILC3s^15,33^. Selective targeting of cDCs or ILC3s, enabled by identification of cell-type-specific genes, demonstrated that neither ILC3s nor dendritic cells, contribute to mucosal tolerance. Whereas the precise ontogeny of Thetis cells remains to be established, a notable finding was their expression of transcription factors and markers typically associated with mTECs. A recent study highlighted the existence of hybrid cell types that emerge from AIRE^+^ mTECs in the thymus^35^. Our discovery of Thetis cells further challenges the current view of boundaries between cell lineages highlighting shared transcriptional programmes between haematopoietic and non-haematopoietic cells that may support common purposes. Within the thymus, AIRE^+^ mTECs instruct T cell tolerance through negative selection and neonatal thymic T_reg_ generation^1,22^. The essential role of Thetis cells in neonatal pT_reg_ differentiation highlights the symmetry between thymic and peripheral tolerance pathways. The expression of AIRE by TC I and TC III suggests the possibility that Thetis cells may share additional functions with mTECs, such as tolerance to self-antigens.

Here we have identified a novel tolerogenic APC type that is enriched in the intestine during a critical early life period when host–microbiota symbiosis is first established. The finding that TC IV instructs extra-thymic T_reg_ cell generation provides a cellular basis for the reported early life window for the establishment of intestinal immune tolerance. Future exploration of Thetis cell biology may yield key insights into mechanisms of immune tolerance and the pathogenesis of autoimmune and inflammatory disease.

## Methods

### Mice

*Rorc*^*Venus-T2A-creERT2*^ mice were generated by insertion of a targeting construct into the *Rorc* 3-UTR by homologous recombination in embryonic stem (ES) cells on the C57Bl/6 background. The IRES-Venus-T2A-creER-frt-NeoR-frt cassette targeting construct was created by cloning. Homologous arms were retrieved from BAC clone RP24-209K20. To facilitate ES cell targeting, the CRISPR–Cas9 system was used. The guide RNA was transcribed in vitro using the MEGA shortscript T7 kit (Life Tech Corp, AM1354) using recombineering techniques. The targeting vector, Cas9 protein (Fisher Scientific, A36498 Truecut Cas9 Protein v2) and guide RNA were co-electroporated into G1 ES cells derived from an F_1_ hybrid blastocyst of 129S6 × C57BL/6J. The resulting chimeras were bred with FLPeR mice to excise the NEO cassette. *Rag1*^*RFP-creERT2*^ (C57BL/6-Tg(Rag1-RFP,-cre/ERT2)33Narl) mice, obtained from the Rodent Model Resource Center, were generated by insertion of a BAC transgene comprising the *Rag1* promoter and RFP-IRES-creERT2 into ES cells from C57Bl/6 mice.

Adig(*Aire*^*GFP*^), *Clec9a*^*cre*^, *Rora*^*cre*^, *Itgb8*^*fl/fl*^, *Cd4*^*cre*^, *H2-Dma*^*–/–*^, C7 and *Itgb8*^*tdTomato*^ mice have been previously described^26,46–52^. *Rorgt*^*cre*^, *H2-Ab1*^*fl/fl*^, *R26*^*lsl-tdTomato*^, *R26*^*lsl-YFP*^, *Zbtb46*^*GFP*^, *Il22*^*cre*^, C57Bl/6 (CD45.2), CD45.1 and BALB/c mice were purchased from Jackson Laboratories. Generation and treatments of mice were performed under protocol 21-05-007 and 08-10-023, approved by the Sloan Kettering Institute (SKI) Institutional Animal Care and Use Committee. All mouse strains were maintained in the SKI animal facility in specific pathogen free conditions in accordance with institutional guidelines and ethical regulations. Both male and female mice were included in the study and we did not observe sex-dependent effects. All mice analysed were age and litter matched unless otherwise specified. All animals used in this study had no previous history of experimentation and were naive at the time of analysis.

### Tamoxifen diet

*Rorc*^*Venus-creERT2*^*H2-Ab1*^*fl/fl*^ and littermate *Rorc*^*Venus-creERT2*^*H2-Ab1*^*fl/fwt*^ mice were placed on a tamoxifen citrate–containing diet (TD.130860; Envigo) at eight weeks of age for five weeks.

### Tissue processing

Mice were euthanized by CO_2_ inhalation. Organs were collected and processed as follows. Lymphoid organs were digested in collagenase in RPMI1640 supplemented with 5% fetal calf serum, 1% l-glutamine, 1% penicillin–streptomycin, 10 mM HEPES, 1 mg ml^−1^ collagenase A (Sigma, 11088793001) and 1 U ml^−1^ DNase I (Sigma, 10104159001) for 45 min at 37 °C, 250 rpm. Large intestine was removed, flushed with PBS and incubated in PBS supplemented with 5% fetal calf serum, 1% l-glutamine, 1% penicillin–streptomycin, 10 mM HEPES, 1 mM dithiothreitol and 1 mM EDTA for 15 min to remove the epithelial layer. Samples were washed and incubated in digest solution for 30 min. Ceramic beads (0.25 inch) (MP Biomedicals, 116540034) were added to large intestine samples (3 per sample) to aid in tissue dissociation. Digested samples were filtered through 100-μm strainers and centrifuged to remove collagenase solution. Thymus samples were minced with scissors followed by enzymatic digestion in RPMI1640 supplemented with 10% fetal calf serum, 1% l-glutamine, 10 mM HEPES, 62.5 μg ml^−1^ Liberase and 0.4 mg ml^−1^ DNase I. Density-gradient centrifugation using a 3-layer Percoll gradient with specific gravities of 1.115, 1.065 and 1.0 was used to enrich for stromal cells for flow cytometric analysis. For sorting of mTECs, single-cell suspension of digested thymocytes were depleted of CD45^+^ cells using CD45 microbeads (Miltenyi Biotec).

### Flow cytometry

For flow cytometric analysis, dead cells were excluded either by staining with LIVE/DEAD Fixable Violet or Zombie NIR in PBS for 10 min at 4 °C, prior to cell-surface staining. Cells were then incubated with anti-CD16/32 in staining buffer (2% FBS, 0.1% Na azide, in PBS) for 10 min at 4 °C to block binding to Fc receptors. Extracellular antigens were stained for 20–30 min at 4 °C or room temperature (CCR7 staining) in staining buffer. For intracellular protein analysis, cells were fixed and permeabilized with Cytofix (BD Biosciences) and/or Ebioscience FOXP3 kit, per manufacturer instructions. Intracellular antigens were stained for 30 min at 4 °C in the respective 1× perm/wash buffer or overnight for intracellular AIRE staining. Live cells were treated with DNase (0.08 U ml^−1^) for 10 min at room temperature and washed with staining buffer prior to acquisition on a BD LSR or Cytek Aurora. 123count eBeads were added to quantify absolute cell numbers. The antibodies used for flow cytometry and FACS are listed in Supplementary Table 3. Unless otherwise stated, we used the following gatings: Thetis cells: Lin (SIGLEC-F, TCRβ, TCRγδ, CD19, B220, NK1.1)^–^CD64^–^Ly6C^–^RORγt (intracellular staining or expression of Venus in *Rorc*^*Venus-creERT2*^ mice) CXCR6^–^MHCII^+^; MHCII^+^ ILC3s: Lin^–^CD64^–^Ly6C^–^RORγt (intracellular staining or expression of Venus in *Rorc*^*Venus-creERT2*^ mice) CXCR6^+^MHCII^+^, and DC2s: Lin^–^CD64^–^Ly6C^–^RORγt^–^CD11c^+^MHCII^+^CD11b^+^.

### Histological analysis of intestinal inflammation

Mice were euthanized by CO_2_ inhalation and large intestines were collected and immediately placed into 10% formalin. Histopathological assessment for inflammation scoring in the intestine was performed on H&E-stained sections based on established scoring systems for intestinal inflammation in mouse models^53^. Assessment includes severity and extent of inflammatory cell infiltrates, epithelial changes and mucosal architecture changes. In brief, the severity and extent of inflammatory cell infiltrates were evaluated histologically. Other evaluations include proliferation of epithelial cells lining the mucosa villous atrophy, crypts, loss of goblet cells, crypt abscesses, erosions and ulceration.

### Multiome scRNA and scATAC-sequencing

For scRNA-seq and scATAC-seq of RORγt^+^MHCII^+^ cells, mLN from 2-week-old (P14–P17) *Rorc*^*Venus-creERT2*^ mice were pooled from 16 biological replicates and processed as described earlier. Cells were depleted of Lin^+^ (TCRb, TCRγδ, CD19, B220, NK1.1)^+^ cells by staining with biotinylated antibodies followed by magnetic bead negative selection. Cells were incubated with anti-CD16/32 in sorting buffer (2% FBS in PBS) for 10 min at 4 °C to block binding to Fc receptors. Extracellular antigens were stained for 30 min at 4 °C in sorting buffer (2% FBS, 2mM EDTA, in PBS). Cells were washed and resuspended in sorting buffer with SYTOX blue (Invitrogen) for exclusion of dead cells. Live, CD45^+^Lin(SIGLEC-F, TCRβ, TCRγδ, CD19)^–^RORγt(Venus)^+^ MHCII^+^ cells were then sort purified. Cells were sorted into cRPMI, before being pelleted and resuspended in RPMI-2% FBS. Single-cell multiome ATAC and gene expression analysis was performed with the 10X genomics system using Chromium Next GEM Single Cell Multiome Reagent Kit A (catalogue no. 1000282) and ATAC Kit A (catalogue no. 1000280) following the user guide for the Chromium Next GEM Single Cell Multiome ATAC + Gene Expression Reagent Kits and the demonstrated protocol for nuclei isolation for single-cell multiome ATAC and gene expression sequencing. In brief, >50,000 cells (viability 95%) were lysed for 4 min and resuspended in Diluted Nuclei Buffer (10x Genomics, 2000207). Lysis efficiency and nuclei concentration was evaluated on Countess II automatic cell counter by trypan blue staining. Nuclei (9,660 per transposition reaction) were loaded, targeting recovery of 6,000 nuclei after encapsulation. After the transposition reaction, nuclei were encapsulated and barcoded. Next-generation sequencing libraries were constructed following the manufacturer’s instructions, and were sequenced on an Illumina NovaSeq 6000 system.

### Plate-based Smart-seq2 sequencing

RORγt^+^MHCII^+^ cells were enriched from a pool of mLN from three-week-old (P21) *Rorc*^*Venus-creERT2*^ mice. Cells were depleted of Lineage (TCRβ, TCRγδ, CD19, B220, NK1.1)^+^ cells via staining with biotinylated antibodies followed by magnetic bead negative selection. Live, Lin(CD3, TCRβ, TCRγδ CD19, B220, NK1.1)^–^CD64^–^Ly6C^–^MHCII^+^RORγt(Venus)^+^ cells were then sorted into single wells. Cells were stained for CD90, CD11c and CD11b for acquiring index-sorting information on cell-surface expression and equal numbers of CD90^–^CD11c^+^, CD90^–^CD11c^–^, CD90^int^ and CD90^hi^ cells were targeted for sorting to ensure representation of all cell types. AIRE^+^ mTECs were enriched from a pool of thymi from three-week old mice via staining with biotinylated antibodies against CD45 followed by magnetic bead negative selection. CD45^–^Epcam^+^MHCII^+^AIRE(GFP)^+^ cells were sorted into single wells. AIRE^+^ dendritic cells were enriched from a pool of MLN from the same three-week old mice. Cells were depleted of Lin^+^ cells as described above and live, Lin(CD3, TCRβ, TCRγδ CD19, B220, NK1.1)^–^CD90^–^CD64^–^Ly6C^–^CD11c^+^MHCII^+^AIRE(GFP)^+^ cells were then sorted into single wells. Retrospective index-sorting analysis confirmed that AIRE(GFP)^+^ cells were CD11c^lo^MHCII^hi^, representing CCR7^+^ dendritic cells.

Single cells were sorted into Buffer RLT (Qiagen). Cell lysates were immediately sealed and spun down before transferring to dry ice and storing at −80 °C. RNA was purified using the Agencourt RNAClean XP beads (Beckman Coulter) at a 2.2× ratio. First-strand cDNA synthesis was achieved using Maxima H Minus Reverse Transcriptase (ThermoFisher) according to the manufacturer’s protocol using oligo dT primers, with the addition of a custom template-switch oligo in a 1 mM final concentration. cDNA was amplified for 24 cycles using KAPA HiFi HotStart ReadyMix (Kapa Biosystems KK2601). After PicoGreen quantification, 0.1–0.2 ng of cDNA was used to prepare libraries with the Nextera XT DNA Library Preparation Kit (Illumina) in a total volume of 6.25 µL with 12 cycles of PCR. Indexed libraries were pooled by volume and cleaned by aMPure XP beads (Beckman Coulter) at a 1× ratio. Pools were sequenced on a HiSeq 4000 in a PE50 or PE100 run using the HiSeq 3000/4000 SBS Kit (Illumina). An average of 1.8 million paired reads were generated per sample and the percent of mRNA bases per sample averaged 63%.

### Mouse scRNA-seq and scATAC-seq computational analysis

#### Pre-processing of the 10X multiome scRNA-seq and scATAC-seq for RORγt^+^MHCII^+^ cells

scRNA-seq and scATAC-seq FASTQ files were aligned to mm10 (Cell Ranger mouse reference genome mm10-2020-A-2.0.0) and counted by Cell Ranger ARC v2.0.0 with default parameters. The barcodes were filtered based on the number of RNA-seqtranscripts (>1,000 and < 50,000), the number of detected genes (>500 and < 6,000), and the fraction of mitochondrial transcripts (<15%). Barcodes were further filtered based on the number of scATAC-seq fragments (3.5 < log_10_(number of fragments) < 4.5) and transcription start site enrichment score (>4). Arrow files were created from the scATAC-seq fragments using ArchR v1.0.1^54^, and doublets were identified and removed with default parameters. Finally, any genes detected in <2 cells in the scRNA-seq data were discarded, leaving 20,779 genes. After clustering, the scRNA-seq data (described in ‘Dimensionality reduction, cell clustering, and visualization’), and based on the expression of marker genes, we identified 5 minor contaminant clusters (glial cells; cluster 17, plasmacytoid dendritic cell; cluster 18, *Rorc*^–/lo^ CCR7^+^ dendritic or Thetis cell; cluster 19, mixed monocyte/cDC1; cluster 20, and macrophage; cluster 21) which were excluded from downstream analyses. In total, 10,145 cells remained, with a median scRNA-seq library size of 3,150 and a median of 13,885 scATAC-seq fragments.

#### Pre-processing of the Smart-seq2 scRNA-seq dataset

Smart-seq2 sequencing data from demultiplexed samples was aligned to the mouse reference genome using STAR v2.7.7a^55^ with ‘--twopassMode Basic --outFilterMultimapNmax 1 --quantMode TranscriptomeSAM’. Sequence reads were aligned and annotated using a STAR index created from GENCODE GRCm38 (mm10) release M25 primary assembly genome and gene annotations^56^. Alignment files were individually name-sorted using Samtools v1.11^57^, and then used to create a cell-by-gene count matrix using featureCounts^58^ (subread v2.0.1). The count matrix was filtered based on the number of transcripts (>50,000), number of detected genes (>1,300), and the fraction of mitochondrial transcripts (<8%). Finally, genes detected in <2 cells were discarded. A total of 481 cells remained, with a median library size of 924,319 from 27,195 genes.

#### Dimensionality reduction, cell clustering, and visualization

For each scRNA-seq dataset, the filtered count matrix was library-size-normalized, log-transformed (‘log-normalized’ expression values) and then centred and scaled (‘scaled’ expression values) using Seurat v4.0.4. Principal component analysis (PCA) was performed on the scaled data (total number of principal components = 50). PhenoGraph clustering^59^ was performed using the first *N* principal components with *k*-nearest neighbours (*N* = 30 and *k* = 30 for the multiome scRNA-seq data; *N* = 20 and *k* = 30 for the Smart-seq2 dataset; *N* = 30 and *k* = 20 for the human gut dendritic cells). Cell clustering was visualized using UMAP^60^, computed from the nearest neighbour graph built by PhenoGraph.

The multiome scATAC-seq data analysis was restricted to the cells in clusters 1–16 of the scRNA-seq results, as previously described for pre-processing. Latent Semantic Indexing (LSI) was performed on 100,000 top variable tiles (500 bp genomic bins) identified after ten iterations of ‘IterativeLSI’ by ArchR. Tiles from non-standard chromosomes, chrM, and chrY were not included in this analysis. Cells were clustered (method=Seurat, k.param = 30, resolution = 1.2) and visualized with UMAP (nNeighbors = 30) using 30 LSI components. In both the scRNA-seq and scATAC-seq data, we identified several clusters of LTi cells (scRNA clusters 9–16 and scATAC clusters 7–13). These clusters showed weak pairwise matchings between scRNA and scATAC; therefore, they were combined as one group of LTi cells for downstream analyses.

#### Differential gene expression tests

DEGs between groups of cells were identified with MAST^61^, performed using Seurat functions. MAST was run on the log-normalized expression values. In all tests, genes were only considered if they were detected in at least 10% of the cells in at least one of the two groups compared (min.pct = 0.1, logfc.threshold = 0). In one-vs-rest differential expression tests comparing multiple groups, each group was compared to all the cells from other groups. Specific differential expression comparisons are described in the results. DEGs were reported according to their fold change (>1.5) and adjusted *P *value (<0.01). Ribosomal and mitochondrial genes were removed from the final list of genes reported or visualized. Where stated, the top DEG markers were subsequently selected for each group, based on fold change.

#### Data imputation for scRNA-seq data

MAGIC imputation^62^ was applied to the log-normalized expression values for the multiome scRNA-seq dataset to further de-noise and recover missing values. Imputed gene expression values were only used for data visualization on UMAP overlays and heatmaps, where stated.

#### Cell cycle scores

Using standard Seurat functions, we computed cell cycle scores for known S-phase and G2/M-phase marker genes^63^ to identify proliferating cells.

#### Topic modelling for scRNA-seq data

Topics were identified by fitting a latent Dirichlet allocation model, also known as a Grade of Membership (GoM) model^64^, to the raw gene expression count matrix for Thetis cells (clusters 1–5 of the multiome scRNA-seq data) using CountClust v1.18.0^65^. Genes that were detected in fewer than 10 Thetis cells were not included. The optimal number of topics (*K* = 8) was selected among values ranging from 3 to 15 with the maximum Bayes factor (BF). The role of a topic in each cell is measured by the degree to which it represents that topic, and the topic weights sum up to 1 in each cell. The importance of a gene for each topic is measured by how distinctively differentially expressed it is in that topic, by measuring the KL-divergence of its relative gene expression to other topics, assuming a Poisson distribution. One topic, defined by ribosomal and mitochondrial genes and shared across all clusters, was removed from the topic model visualizations.

#### Dynamical modelling of RNA velocity for the multiome scRNA-seq data

The unspliced and spliced mRNAs for the scRNA-seq profiles of the multiome data were counted by Velocyto v0.17.17^32^ from the position-sorted BAM file containing GEX read alignments, outputted by Cell Ranger ARC in pre-processing. As annotation files for Velocyto, we used the same mm10 gene annotations used in pre-processing, in addition to the mm10 expressed repeat annotation from the RepeatMasker track of UCSC genome browser. Next, we used the Velocyto results to learn a generalized dynamical model of RNA velocities by scVelo v0.2.4^66^. Count matrices were filtered, normalized, and log-transformed (min_shared_counts = 10, n_top_genes = 3000), cell cycle effect was corrected by regressing out S-phase and G2/M-phase scores, using Scanpy 1.6.0^67^. After performing PCA on the corrected data (n_pcs = 30), first- and second-order moments were computed for each cell across its nearest neighbours in the PCA space (n_neighbors = 30). Finally, the full splicing kinetics were recovered and solved for each gene by scVelo’s dynamical model.

#### Integrating the Smart-seq2 dataset with the multiome dataset

RORγt^+^MHCII^+^ transcriptomes (based on cell-type as sorted) from the SMART-seq2 dataset were integrated with transcriptomes from the 10X multiome scRNA-seq data, using Seurat^68^. Based on the variability of genes in both datasets, 5,000 top scoring genes were selected by Seurat functions to identify ‘integration anchors’ with canonical correlation analysis (CCA). Expression values for these genes were integrated, scaled, and used for PCA. A UMAP embedding was computed from the first *N* = 30 principal components (*k* = 30). Additionally, using Seurat functions, the RORγt^+^MHCII^+^ cells from the SMART-seq2 dataset (query) were mapped to multiome scRNA-seq clusters (reference) by projecting the PCA from the reference onto the query to identify ‘transfer anchors’, and then assigning a prediction score for each reference cluster to query cells. The cluster identity with the highest score was chosen as the predicted label for each cell.

#### Single-cell enrichment scores for gene sets

Given a set of genes, we standardized the log-normalized expression values of each gene across cells and then averaged these values for all genes in the set, assigning an enrichment score to each cell. Where stated, these scoreswere standardized across cells and reported as *z*-scores.

#### Creating pseudo-bulk samples from scRNA-seq data

Pseudo-bulk samples were created by averaging the unimputed log-normalized gene expression values for each cluster. In cases where scaled values were used for downstream analyses, these average expression values were standardized across the pseudo-bulk samples.

#### Similarity of multiome scRNA-seq clusters to bulk microarray ImmGen samples

The RMA-normalized and log_2_-transformed gene expression data of 224 bulk microarray samples from a publicly availableImmGen dataset was downloaded from https://www.haemosphere.org^69^. For each gene, the probeset with the highest mean expression was retained. We included all cell types isolated from naive, untreated mice. Pseudo-bulk samples were generated from the multiome scRNA-seq data for each Thetis cell subset, and non-proliferating MHCII^+^ ILC3s (NCR^+^ ILC3 and LTi cells). The gene expression vectors were scaled across bulk and pseudo-bulk samples within each dataset, and their pairwise cosine similarities were used to compare the samples. These similarity scores were computed from the expression of 2,399 DEGs (FC > 1.3, adjusted *P* < 0.01) comparing the scRNA-seq clusters in a one-vs-rest test, that were also expressed in the microarray data. The proliferating and progenitor clusters were excluded from the differential expression test, and the LTi clusters were grouped together. For visualization of results, only cell lineages containing a cell type with > 0.25 cosine similarity with either Thetis cell or ILC3 clusters were plotted.

#### Cell-type prediction

To determine similarity between Thetis cells and known cell types we used CellTypist (https://www.celltypist.org), with both low- and high-resolution models of immune cells to classify cells with coarse and fine granularities, respectively. Top predicted labels for each input cell were visualized.

#### Similarity of Thetis cells to thymic epithelial cells

scRNA-seq profiles of CD45^–^ thymic epithelial cells were downloaded from a publicly available dataset (GSE103967)^21^. The raw counts were library-size-normalized, log-transformed, and used to create pseudo-bulk samples for each thymic epithelial cluster. Pseudo-bulk samples were also generated to represent the multiome scRNA-seq Thetis cell clusters (2–5). These pseudo-bulk gene expression vectors were scaled across samples within each dataset, and their pairwise cosine similarities were used to compare clusters from the two datasets. These similarity scores were computed from the expression of 1,740 DEGs (FC > 1.3, adjusted *P* < 0.01) identified in a one-vs-rest differential expression test for non-proliferating Thetis cell clusters (2–5), that were also expressed in the thymic epithelial cells. Among individual clusters of thymic epithelial cells defined in the original dataset, we identified 2 clusters of transit amplifying AIRE^+^ cells (clusters 25 and 26), distinguished by signature gene expression including cell cycle genes.

#### scRNA-seq dataset of human gut dendritic cells

Dendritic cells (annotated as cDC1, cDC2 or lymphoid DC) within the myeloid dataset from the human gut atlas^41^ were re-clustered. From the gene markers for each Thetis cell subset (one-vs-rest differential expression test for non-proliferating Thetis cell scRNA-seq clusters, FC > 1.5, adjusted *P* < 0.01), we identified orthologous human genes that were uniquely mapped by gprofiler2 and computed enrichment scores for Thetis cell subset gene signatures for each human cell.

#### Peak calling for the multiome scATAC-seq data

For peak calling of the scATAC-seq data, clusters for similar cell types were grouped: C1 (TC IV), C2–4 (TC I–TC III), C5–6 (NCR^+^ ILC3), and C7–13 (LTi). Filtered scATAC-seq fragments for each group were extracted from ArchR arrow files. We performed MACS2 v2.2.7.1 on fragments of each group with ‘--gsize mm --qval 0.01 --nomodel --ext 200 --shift −100 --call-summits’. The peak summits were extended by 100 bp in each direction. Regions extending outside of mm10 chromosomes, arising from chrY or chrM, overlapping with blacklist regions precompiled by ArchR (merged from the ENCODE mm10 v2 blacklist regions from https://github.com/Boyle-Lab/Blacklist/blob/master/lists/mm10-blacklist.v2.bed.gz and mitochondrial regions that are highly mappable to the mm10 nuclear genome from https://github.com/caleblareau/mitoblacklist/blob/master/peaks/mm10_peaks.narrowPeak), or containing ‘N’ nucleotides (>0.001 of the sequence) were filtered. Regions from all groups were compiled and overlapping regions were merged to their union, resulting in a non-overlapping set of 176,942 peaks. A peak-by-cell count matrix was created by ArchR with a ‘ceiling’ value of 4 for the counts to avoid strong biases.

#### Transcription factor motif enrichment with chromVAR

The peaks that were accessible in <10 cells were filtered from the peak insertion counts, created as described in the previous section, and the resulting 176898 x 10145 peak-by-cell count matrix was used for motif enrichment with chromVAR v1.14.0^70^. Mouse motif PWMs were downloaded from the CIS-BP database^71^ (‘Mus_musculus_2022_01_14_6-40_pm’), and the missing PWMs were extracted from ‘mouse_pwms_v1’ in chromVARmotifs v0.2.0. The GC content of the peaks was computed with chromVAR, and motifs were matched to them by motifmatchr v1.14.0. Then, chromVAR ‘deviations’ of the motifs were computed for the peak-by-cell count matrix. The ‘top motif’ for each transcription factor was selected by correlating its log-normalized gene expression values (from multiome scRNA-seq) with the deviation *z*-scores of its motifs, in the same cells, and picking the motif with the highest Pearson correlation coefficient. Finally, transcription factor–motif pairs with a correlation higher than 0.1 were selected. This resulted in 56 top transcription factors, out of 739 CIS-BP transcription factors that were expressed (that is, had any transcripts detected) in the multiome scRNA-seq profiles. The same process was repeated for the 139,528 × 1,552 peak-by-cell count matrix of Thetis cells (multiome scATAC-seq clusters 1–4) and the peaks accessible in at least 10 Thetis cells. Out of 652 CIS-BP transcription factors that were expressed in Thetis cells, 68 had a transcription factor–motif correlation higher than 0.1 and were selected as top transcription factors for Thetis cells.

### Neonatal 4-OH tamoxifen administration

For labelling of RORγt^+^ cells, *Rorc*^*Venus-creERT2*^*Aire*^*GFP*^ mice were injected intraperitoneally on P1 with 25 μg 4-OH-tamoxifen (4-OHT) and analysed on P8, P15 and P21. For RAG1 fate mapping, *Rag1*^*RFP-creERT2*^*R26*^*lsl-YFP*^ mice were injected with 25 μg 4-OHT intraperitoneally on P3, P5 and P7 and analysed on P15.

### Electron microscopy

RORγt^+^MHCII^+^ cells were enriched from a pool of mLN from P18 *Rorc*^*Venus-creERT2*^ mice (for TC IV or reference CCR7^–^ and CCR7^+^ dendritic cells) and P18 *Aire*^*GFP*^ mice (for TC I). Cells were depleted of Lin^+^ (TCRb, TCRgd, CD19, B220, NK1.1)^+^ cells via staining with biotinylated antibodies followed by magnetic bead negative selection. mTECs were enriched from a pool of thymi from P18 *Aire*^*GFP*^ mice as described above. Live, Lin(TCRb, TCRgd, CD19, B220, NK1.1)^–^CD64^–^Ly6C^–^MHCII^+^RORγt(Venus)^+^CD11c^+^CD11b^+^cells (TC IV), Lin^–^RORγt(Venus)^–^CD11c^lo^MHCII^hi^ (CCR7^+^ dendritic cell), Lin^–^RORγt(Venus)^–^CD11c^hi^MHCII^–^ (CCR7^–^ dendritic cell), Lin^–^CXCR6^–^CD11c^–/lo^AIRE(GFP)^hi^ (TC I), Lin^–^RORγt(Venus)^+^CD90^+^MHCII^+^ (LTi/ILC3), or CD45^–^Epcam^+^MHCII^+^AIRE(GFP)^+^ cells were then sorted directly into 2% glutaraldehyde, 4% PFA, and 2 mM CaCl_2_ in 0.1 M sodium cacodylate buffer (pH 7.2), fixed for > 1 h at room temperature, postfixed in 1% osmium tetroxide, dehydrated in acetone, and processed for Epon embedding. Ultrathin sections (60–65 nm) were counterstained with uranyl acetate and lead citrate. Images were taken with a transmission electron microscope (Tecnai G2–12; FEI) equipped with a digital camera (AMT BioSprint29).

### Tissue preparation for immunofluorescence, microscopy and image analysis

Mesenteric lymph nodes (mLN) were dissected from 2- to 3-week-old *Rorc*^*Venus-creERT2*^ or *Aire*^*GFP*^ mice and trimmed of fat using a dissection scope and forceps. mLNs were fixed in 2% paraformaldehyde for 4 h in 4 °C, washed 3 times in PBS, and dehydrated in 30% sucrose in 0.1 M phosphate buffer overnight (16–20 h). mLNs were embedded in optimal cutting temperature (OCT) compound, frozen on dry ice, and stored at −80 °C. 15–20m sagittal sections were placed on Superfrost Plus microscopy slides and stored at −20C until staining. mLN sections were permeabilized using 0.2% Triton X-100 for 15 min at room temperature, washed 3 times with PBS, blocked in 5% rabbit and donkey serum for 1 h at room temperature, and washed 3 times with PBS. Next, the sections were incubated with combinations of the following primary antibodies in PBS overnight at 4 °C: CD11c BV421 (Biolegend, Clone N418, 1:50), CD11b BV480 (BD Biosciences, Clone M1/70 1:50) CD4 AF647 (Biolegend, Clone RM4-5, 1:50), FOXP3 eFluor 570 (ThermoFisher, Clone FJK-16s, 1:50), GFP AF488 (ThermoFisher A12311, polyclonal, 1:100), RORγt APC (ThermoFisher, Clone AFKJS-9, 1:50), and MHCII AF700 (Biolegend, Clone M5/114.15.2, 1:200) antibodies. The samples were washed three times in PBSthe next day and mounted in SlowFade Diamond antifade reagent (ThermoFisher). No. 1.5 coverglass was used to seal the slide and all subsequent imaging was done on Leica SP8 microscope. Analysis was performed by histo-cytometry methods using Imaris software. Image segmentation was performed in Imaris using the ‘Surface object creation’ module, which uses a seeded region growing, *k*-means, and watershed algorithm to define individual cells.

### In vitro cell culture

Naive CD4^+^vβ10^+^CD25^–^CD44^lo^CD62L^hi^ C7 T cells were sort purified after enrichment with a CD4^+^ T cell negative selection kit (Miltenyi Biotec). Dendritic and Thetis cell subsets were sort purified from *Rorc*^*Venus-creERT2*^ mice using gating strategy above with inclusion of CCR6 to distinguish TC I and TC II from TC III and TC IV. cDC2s were distinguished from their CCR7^+^ counterparts by CD11c^hi^MHCII^int^ expression. T cells were co-cultured in triplicate at a ratio of 300 dendritic or Thetis cells to 1 × 10^3^ T cells in the presence of ESAT peptide (1 µg ml^−1^; Invivogen), with the addition of 0.5 ng ml^−1^ TGFβ1 (Peprotech), 100 IU ml^−1^ of IL-2 (NCI). FOXP3 expression was assessed after 4 days of culture.

### Bone marrow chimeric mice

Bone marrow cells were isolated from indicated donor mice, depleted of CD90.2^+^ and TER-119^+^ cells using magnetic bead based depletion. Bone marrow cells were resuspended in PBS and 2–3 × 10^6^ cells were injected into 6-week-old CD45.1 mice, irradiated with 950 rad per mouse 1 day earlier. After 6 weeks, mLN and LI LP were collected for analysis.

### Statistical analysis

Analysis of all data was done with unpaired two-tailed *t*-test, one or two-way ANOVA with a 95% confidence interval, or Mann–Whitney *U* test, as specified in the text or figure legends. *P* < 0.05 was considered significant: *P < 0.05, **P < 0.01, ***P < 0.001, ****P < 0.0001. Details of number of replicates, sample size, significance tests and value and meaning of *n* for each experiment are included in the Methods or figure legends. Statistical tests were performed with Prism (GraphPad Software). scATAC and scRNA-seq experiments were carried out once. Mice were non-randomly allocated to experimental groups to ensure equal distribution of genotypes between treatments. Researchers were not blinded to genotype or treatment during the experiments. No measures were taken to estimate sample size of to determine whether the data met the assumptions of the statistical approaches used. Significance (*α*) was defined as <0.05 throughout, after correcting for multiple comparisons.

### Reporting summary

Further information on research design is available in the Nature Research Reporting Summary linked to this article.

## Online content

Any methods, additional references, Nature Research reporting summaries, source data, extended data, supplementary information, acknowledgements, peer review information; details of author contributions and competing interests; and statements of data and code availability are available at 10.1038/s41586-022-05309-5.

## Supplementary information

Reporting Summary

Supplementary Table 1 Genes differentially expressed between mTECs and Thetis cells (SS2).

Supplementary Table 2 Differential gene expression for SS2 Thetis cell and ILC3 clusters.

Supplementary Table 3 List of antibodies.

## Data Availability

The mouse sequencing data are available through the Gene Expression Omnibus under accession number GSE174405. This manuscript makes use of publicly available data from the Human Cell Atlas, available at https://www.gutcellatlas.org. Source data are provided with this paper.

## Source data

Source Data Fig. 1
Source Data Fig. 3
Source Data Fig. 4
Source Data Fig. 5
Source Data Extended Data Fig. 1
Source Data Extended Data Fig. 2
Source Data Extended Data Fig. 3
Source Data Extended Data Fig. 4
Source Data Extended Data Fig. 5
Source Data Extended Data Fig. 6
Source Data Extended Data Fig. 8
Source Data Extended Data Fig. 9
Source Data Extended Data Fig. 10
