# Supplementary material for: Novel antigen-presenting cell imparts Treg-dependent tolerance to gut microbiota
Source: Nature. 2022 Sep 7;610(7933):752–60. doi: 10.1038/s41586-022-05309-5 (PMC9605865; doi:10.1038/s41586-022-05309-5)
Supplement: Supplementary file 1 — Reporting Summary [file 41586_2022_5309_MOESM1_ESM.pdf]

nature portfolio

Corresponding author(s): Chrysothemis Brown and Alexander Rudensky

Last updated by author(s): Jun 30, 2022

Reporting Summary

Nature Portfolio wishes to improve the reproducibility of the work that we publish. This form provides structure for consistency and transparency in reporting. For further information on Nature Portfolio policies, see our Editorial Policies and the Editorial Policy Checklist.

Statistics

For all statistical analyses, confirm that the following items are present in the figure legend, table legend, main text, or Methods section.

n/a	Confirmed	
☐	☒	The exact sample size (*n*) for each experimental group/condition, given as a discrete number and unit of measurement
☐	☒	A statement on whether measurements were taken from distinct samples or whether the same sample was measured repeatedly
☐	☒	The statistical test(s) used AND whether they are one- or two-sided<br>*Only common tests should be described solely by name; describe more complex techniques in the Methods section.*
☒	☐	A description of all covariates tested
☐	☒	A description of any assumptions or corrections, such as tests of normality and adjustment for multiple comparisons
☐	☒	A full description of the statistical parameters including central tendency (e.g. means) or other basic estimates (e.g. regression coefficient) AND variation (e.g. standard deviation) or associated estimates of uncertainty (e.g. confidence intervals)
☐	☒	For null hypothesis testing, the test statistic (e.g. *F*, *t*, *r*) with confidence intervals, effect sizes, degrees of freedom and *P* value noted<br>*Give P values as exact values whenever suitable.*
☒	☐	For Bayesian analysis, information on the choice of priors and Markov chain Monte Carlo settings
☒	☐	For hierarchical and complex designs, identification of the appropriate level for tests and full reporting of outcomes
☒	☐	Estimates of effect sizes (e.g. Cohen's *d*, Pearson's *r*), indicating how they were calculated

Our web collection on statistics for biologists contains articles on many of the points above.

Software and code

Policy information about availability of computer code

Data collection	Cytek Aurora and Facs Diva (BD Biosciences) for flow cytometry. 10X Chromium for single cell RNA and ATAC-sequencing. Details for RNA-seq and ATAC-seq are included in the methods.
Data analysis	Flowjo v10 for flow cytometry data. GraphPad Prism v9 and v10 for statistical analyses. All computational methods are detailed in the methods section and code is available upon request.

For manuscripts utilizing custom algorithms or software that are central to the research but not yet described in published literature, software must be made available to editors and reviewers. We strongly encourage code deposition in a community repository (e.g. GitHub). See the Nature Portfolio guidelines for submitting code & software for further information.

Data

Policy information about availability of data

All manuscripts must include a data availability statement. This statement should provide the following information, where applicable:

- Accession codes, unique identifiers, or web links for publicly available datasets
- A description of any restrictions on data availability
- For clinical datasets or third party data, please ensure that the statement adheres to our policy

The mouse sequencing data are available through the Gene Expression Omnibus under accession GSE174405. Sequencing data for the Human Gut Atlas has been published previously and the processed data is publicly available from https://www.gutcellatlas.org

Human research participants

Policy information about studies involving human research participants and Sex and Gender in Research.

Reporting on sex and gender	N/A
Population characteristics	N/A
Recruitment	N/A
Ethics oversight	N/A

Note that full information on the approval of the study protocol must also be provided in the manuscript.

Field-specific reporting

Please select the one below that is the best fit for your research. If you are not sure, read the appropriate sections before making your selection.

☒ Life sciences ☐ Behavioural & social sciences ☐ Ecological, evolutionary & environmental sciences

For a reference copy of the document with all sections, see nature.com/documents/nr-reporting-summary-flat.pdf

Life sciences study design

All studies must disclose on these points even when the disclosure is negative.

Sample size	No statistical methods were used to determine sample size. Sample size was determined based on similar experiments. In most experiments 3-6 mice per group were used, with exact numbers and statistical tests details in the figure legends and methods section.
Data exclusions	No data exclusions.
Replication	Replicate experiments performed with number of replicate experiments (minimum of 2) and biological replicate samples detailed in the accompanying figure legends or methods.
Randomization	Mice were randomly assigned for experiments reported. Both male and female age-matched littermate controls were used for experiments.
Blinding	Investigators were not blinded to group allocations during the experiment or data analysis.

Reporting for specific materials, systems and methods

We require information from authors about some types of materials, experimental systems and methods used in many studies. Here, indicate whether each material, system or method listed is relevant to your study. If you are not sure if a list item applies to your research, read the appropriate section before selecting a response.

Materials & experimental systems

n/a	Involved in the study	
☐	☒	Antibodies
☒	☐	Eukaryotic cell lines
☒	☐	Palaeontology and archaeology
☐	☒	Animals and other organisms
☒	☐	Clinical data
☒	☐	Dual use research of concern

Methods

n/a	Involved in the study	
☒	☐	ChIP-seq
☐	☒	Flow cytometry
☒	☐	MRI-based neuroimaging

Antibodies

Antibodies used	All antibodies used are listed in a supplementary table.
Validation	All commercially available antibodies are routinely tested by the vendor.

Animals and other research organisms

Policy information about studies involving animals; ARRIVE guidelines recommended for reporting animal research, and Sex and Gender in Research

Laboratory animals	pecies and strain information are reported in the methods section. Mice were used at specified ages as detailed in text and figure legends. Mice within each experiment are age and sex matched.
Wild animals	N/A
Reporting on sex	Both male and females were used in the study and we did not observe any sex-specific phenotypes.
Field-collected samples	N/A
Ethics oversight	Animal procedures were approved and performed according to the IACUC at Memorial Sloan Kettering Cancer Center

Note that full information on the approval of the study protocol must also be provided in the manuscript.

Flow Cytometry

Plots

Confirm that:

☒ The axis labels state the marker and fluorochrome used (e.g. CD4-FITC).

☒ The axis scales are clearly visible. Include numbers along axes only for bottom left plot of group (a 'group' is an analysis of identical markers).

☒ All plots are contour plots with outliers or pseudocolor plots.

☒ A numerical value for number of cells or percentage (with statistics) is provided.

Methodology

Sample preparation	Sample preparation was performed as described in the methods section
Instrument	BD Aria for cell sorting, BD LSR or Cytek Aurora for flow cytometric analysis.
Software	Flowjo v10
Cell population abundance	Frequencies of cell populations are indicated on the flow plots.
Gating strategy	A detailed gating strategy is included in the supplementary methods section.

☒ Tick this box to confirm that a figure exemplifying the gating strategy is provided in the Supplementary Information.
